# Central nervous system antituberculosis drug exposures in a rabbit model of tuberculous meningitis

**DOI:** 10.1128/aac.00261-26

**Published:** 2026-07-10

**Authors:** Jose M. Calderin, Sean Wasserman, Rosleine Antilus-Sainte, Melissa Cristaldo, Narineh M. Odjourian, Faye Lanni, Firat Kaya, Noha Abdelgawad, Juan Eduardo Resendiz-Galvan, Matthew Zimmerman, Veronique Dartois, Paolo Denti, Martin Gengenbacher

**Affiliations:** 1 Division of Clinical Pharmacology, Department of Medicine, University of Cape Town 536985 , Observatory, Cape Town, South Africa; 2 Wellcome Discovery Research Platforms in Infection, Centre for Infectious Diseases Research in Africa, Institute of Infectious Disease and Molecular Medicine, University of Cape Town, Observatory, Cape Town, South Africa; 3 Blizard Institute, Queen Mary University of London 105711, London, United Kingdom; 4 Center for Discovery and Innovation, Hackensack Meridian Health, Nutley 464821 , New Jersey, United States; 5 Hackensack Meridian School of Medicine 576909, Nutley, New Jersey, United States; Providence Portland Medical Center 25165, Portland, Oregon, USA

**Keywords:** tuberculous meningitis, central nervous system, cerebrospinal fluid, pharmacokinetics, nonlinear mixed-effects modeling, rabbit model, pyrazinamide, isoniazid, bedaquiline, rifampicin, rifabutin, linezolid

## Abstract

Treatment optimization through pharmacokinetic evaluation is needed to improve outcomes in tuberculous meningitis (TBM). Clinical assessment of drug penetration into the central nervous system (CNS) is limited to cerebrospinal fluid (CSF) sampling, which may not reflect drug exposures at the site-of-disease. We characterized the pharmacokinetics of antituberculosis drugs in CNS tissues using a rabbit model of TBM. Rabbits infected with *Mycobacterium tuberculosis* received human-equivalent doses of antituberculosis drugs after displaying signs of TBM. Blood was intensively sampled on day 1 and until euthanasia on day 3 when terminal plasma, CSF, and CNS tissues were collected. Total drug concentrations were quantified by LC–MS/MS and analyzed in NONMEM to estimate equilibration half-lives and CSF- and CNS tissue-to-plasma ratios. Across 5 experiments, 48 rabbits provided 600 plasma samples and 48 sets of terminal CSF/CNS tissue samples. Pyrazinamide and isoniazid showed high and consistent penetration, with CSF ratios of 0.98 and 0.85 and CNS tissue ratios ranging from 0.44 to 0.73 and 0.60–0.82, respectively. Linezolid demonstrated modest and uniform penetration (CSF ratio 0.39; CNS tissue ratios 0.15–0.44). Rifampicin showed the lowest overall penetration (CSF ratio 0.08; CNS tissue ratios 0.07–0.32). In contrast, the most lipophilic drugs, bedaquiline and rifabutin, showed low CSF ratios (0.01 and 0.42, respectively) compared with much higher CNS tissue ratios (1.11–6.34 and 1.23–6.78, respectively). CSF penetration in the rabbit TBM model closely mirrored reported human data, establishing its translational value. High relative CNS exposures of bedaquiline and rifabutin support their clinical evaluation for TBM treatment.

## INTRODUCTION

Tuberculous meningitis (TBM) leads to mortality in over 40% of patients, with a high proportion of survivors left with chronic neurological impairment (1). One potential reason for these severe outcomes is suboptimal drug exposures at the site of disease. The recommended treatment regimen for TBM is identical to pulmonary TB and not optimized for infection in the central nervous system (CNS) (1), where penetration of antituberculosis drugs is restricted by the blood–brain barrier (BBB) and the blood–cerebrospinal fluid barrier (BCSFB) (1, 2). Moreover, inflammation and disease-related alterations in barrier integrity may further influence the penetration of CNS-targeted drugs (1, 2).

The cerebrospinal fluid (CSF) penetration of several antituberculosis drugs has been previously reported in humans (3–5). Clinical studies showed that pyrazinamide and isoniazid—both small, hydrophilic molecules with low plasma protein binding—achieve CSF exposures comparable to those in plasma (3). In contrast, rifampicin, a key drug in TBM treatment, is a relatively large molecule with high plasma protein binding and exhibits poor CSF penetration, achieving CSF concentrations 10- to 20-fold lower than those in plasma (4, 6). Higher rifampicin doses (>20 mg/kg) may increase CSF exposures and are currently under evaluation in TBM clinical trials (4). Despite its central role in TB treatment, rifampicin is a strong inducer of metabolic enzymes and transporters, including at the BBB (7), potentially affecting the pharmacokinetics of co-administered drugs. On the other hand, rifabutin—a highly lipophilic rifamycin with weaker enzyme-inducing activity and efficacy comparable to rifampicin in pulmonary TB trials (8)—demonstrates good CSF penetration (5), making it an attractive candidate for TBM treatment.

The CSF penetration of second-line TB drugs such as linezolid and bedaquiline has also been explored (9, 10). Linezolid, a Group A drug recommended by the WHO for MDR-TB, has a high unbound plasma fraction and demonstrates moderate penetration into the CSF of TBM patients (11). Bedaquiline, a highly lipophilic diarylquinoline associated with improved outcomes when added to pulmonary MDR-TB regimens, is reported to cross the BBB freely but achieves low CSF concentrations in patients with pulmonary TB, likely due to its extensive plasma protein binding (10).

Direct measurement of drug concentrations in CNS tissues is not feasible in clinical settings. Lumbar CSF concentrations are commonly used as a surrogate for brain concentrations but may not accurately represent drug levels in the brain parenchyma (12). Differences in barrier structure and permeability, transporter expression, and tissue binding between the BBB and BCSFB can contribute to discrepancies between CSF and brain drug concentrations (12, 13). Preclinical models that replicate key aspects of human disease provide a valuable framework for evaluating drug penetration into CNS tissues, offering crucial insights to guide treatment optimization for TBM (14). We aimed to characterize the pharmacokinetics of antituberculosis drugs in CNS tissue using a rabbit model of TBM.

## MATERIALS AND METHODS

### Preclinical TBM model and drug administration

We previously developed a rabbit model optimized to recapitulate key features of human TBM, including variable duration of symptom onset, characteristic clinical manifestations, representative neuropathology, and radiological findings consistent with the human disease (15). Briefly, New Zealand White rabbits were inoculated with 10⁴ CFU of *Mycobacterium tuberculosis* strain HN878 via the cisterna magna and treatment initiated once animals reached a neurological score of 3 (15, 16). Symptomatic rabbits received human-equivalent daily doses of study drugs by oral gavage for 3 consecutive days. Human-equivalent doses were established in prior dose-finding studies in uninfected rabbits to match published human plasma exposures, defined by area under the concentration–time curve (AUC). Dosing details for each experiment are summarized in Table 1, and the formulations of the study drugs are described in Material S1.

**TABLE 1:** Dosing regimens and corresponding human-equivalent doses for each experiment

Experiment	Rabbit dose	Human-equivalent dose	Administration
Pyrazinamide	175 mg/kg	1,500 mg	Co-administered with isoniazid
Isoniazid	30 mg/kg	300 mg	Co-administered with pyrazinamide
Bedaquiline	400 mg	400 mg	Monotherapy
Rifampicin	90 or 120 mg/kg	35 mg/kg	Monotherapy
Rifabutin	15 mg/kg	300 mg	Monotherapy
Linezolid	90 mg/kg	1,200 mg	Monotherapy

### Sample collection and quantification

Blood samples were collected from the central auricular artery on days 1 and 3. On day 3, animals were anesthetized and euthanized at predetermined time points post-dosing. Plasma samples were collected over the full dosing interval on day 1 (0–24 h post-dose) and up to the time of necropsy on day 3. During necropsy, terminal samples were collected from CSF, lumbar and cervical cord, meninges, and brain, as well as from extra-CNS tissues including the lung, liver, and spleen. Table 2 summarizes the blood sampling schedules and necropsy time points for the study drugs. All animal experiments were reviewed and approved by the Hackensack Meridian Health Institutional Animal Care and Use Committee (Protocol No. 270).

**TABLE 2:** Blood sampling schedule and necropsy time for each experiment

Experiment	Blood sampling schedule^a^	Necropsy time
Pyrazinamide + Isoniazid	Pre-dose, and 0.5, 1, 2, 3, 4, 6, 24 h post-dose	2, 6 h
Bedaquiline	Pre-dose, and 0.5, 2, 4, 6, 24 h post-dose	24 h
Rifampicin	Pre-dose, and 0.5, 1, 2, 3, 5, 7, 10, 24 h post-dose	2, 3, 6, 10, 24 h
Rifabutin	Pre-dose, and 0.5, 1, 2, 3, 4, 5, 6, 7, 10, 24 h post-dose	3, 6, 10, 24 h
Linezolid	Pre-dose, and 0.5, 1, 2, 3, 5, 7, 24 h post-dose	2, 6, 24 h

^a^ Pre-dose samples were collected only on day 3.

Plasma, CSF, and tissue samples were quantified for total drug concentrations (protein-bound plus unbound) using validated liquid chromatography–mass spectrometry assays (Material S2).

### Pharmacokinetic modeling

Nonlinear mixed-effects modeling was performed in NONMEM version 7.5.1 (17) to develop separate population pharmacokinetic models for each study drug, describing plasma, CSF, and CNS tissue concentration-time data. A sequential, non-simultaneous approach was used: plasma concentrations were modeled first, followed by the stepwise incorporation of CSF and CNS tissue data. Individual plasma pharmacokinetic parameters were obtained as *post hoc* empirical Bayes estimates and subsequently fixed to inform the estimation of CSF and CNS tissue penetration parameters (18).

CSF and CNS tissue data were described using hypothetical effect compartments (19), each linked to the central (plasma) compartment. This approach characterizes drug penetration into CSF and CNS tissues by estimating the plasma-to-CSF/tissue equilibration half-life (T½_Plasma–CSF/tissue_) and the CSF/tissue-to-plasma ratio (Ratio_CSF/tissue–Plasma_). These parameters reflect the delay in concentration equilibration between plasma and these compartments, as well as the extent of drug penetration in these compartments relative to plasma at steady state (Material S3). The precision of the parameter estimates in the final models, indicated by 95% confidence intervals, was evaluated through the sampling importance resampling (SIR) method (20).

### Simulations

Concentration–time profiles of the study drugs in plasma, CSF, and CNS tissues were simulated for a typical adult male TB patient (55 kg body weight [WT]) (21). Previously published pharmacokinetic models that included CSF compartments (3, 4, 11), with disposition parameters (clearances and volumes of distribution) allometrically scaled to the reference individual (WT: 55 kg), were used to simulate reference plasma and CSF profiles in humans. For bedaquiline and rifabutin, where no previously published CSF pharmacokinetic models were available, existing plasma models (22, 23) were used to simulate plasma profiles. Reference human CSF profiles were constructed using information from other studies (5) (Upton C, personal communication, July 2025).

First-order plasma-to-CSF/tissue equilibration rate constants estimated in our analysis of rabbit data were scaled across species to the reference individual (WT: 55 kg) using a power-law relationship with WT (exponent: –0.25), as described by Boxenbaum et al. (24*)*. The scaled parameters were then applied to simulate day-3 concentration–time profiles following daily dosing with pyrazinamide (1,500 mg), isoniazid (300 mg), bedaquiline (400 mg), rifampicin (35 mg/kg), rifabutin (300 mg), and linezolid (1,200 mg).

## RESULTS

### Study data

**Fig 1:**
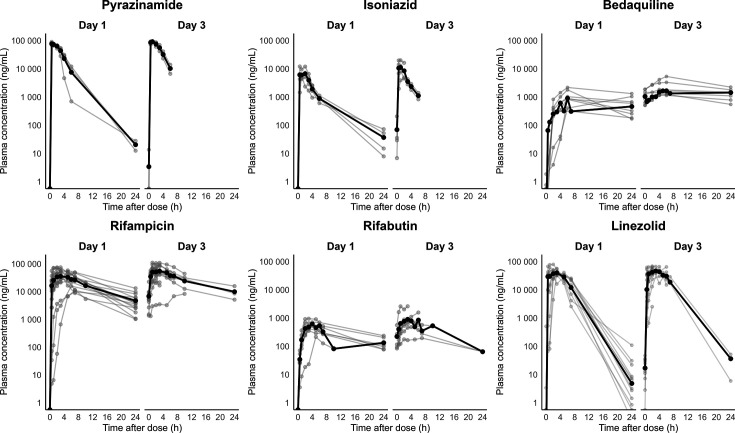
Plasma concentration–time data of pyrazinamide, isoniazid, bedaquiline, rifampicin, rifabutin, and linezolid in rabbits with TBM on 1 and 3 days of dosing. Gray lines indicate individual animal profiles, and black lines represent the median profile for each sampling day. Day 1 profiles were plotted from time 0, although no pre-dose sample was collected before the first dose, and the log-scale *y*-axis is shown from 1 ng/mL to improve visualization. All collected samples were quantifiable, except for three pyrazinamide pre-dose samples on day 3.

Plasma, CSF, and tissue samples were collected from rabbits after 3 days of once-daily dosing with the study drugs (Table 3). All collected plasma samples were quantifiable for all drugs except pyrazinamide, for which three pre-dose samples on day 3 were below the limit of quantification (BLQ; LLOQ: 1 ng/mL). Plasma data from each experiment are presented in Fig. 1.

**TABLE 3:** Number of animals and collected samples per experiment^*a*^

Experiment	Number of animals	Weight (kg)	Plasma samples	Paired terminal samples (plasma, CSF, tissue)
Pyrazinamide + Isoniazid	4	2.9 (2.7–3.1)	50	4
Bedaquiline	8	3.0 (2.3–3.4)	84	8
Rifampicin	16	3.0 (2.4–3.2)	215	16
Rifabutin	7	3.2 (2.8–4.4)	100	7
Linezolid	13	4.1 (3.0–4.7)	151	13

^*a*^ Data are presented as n or median (min–max).

**Fig 2:**
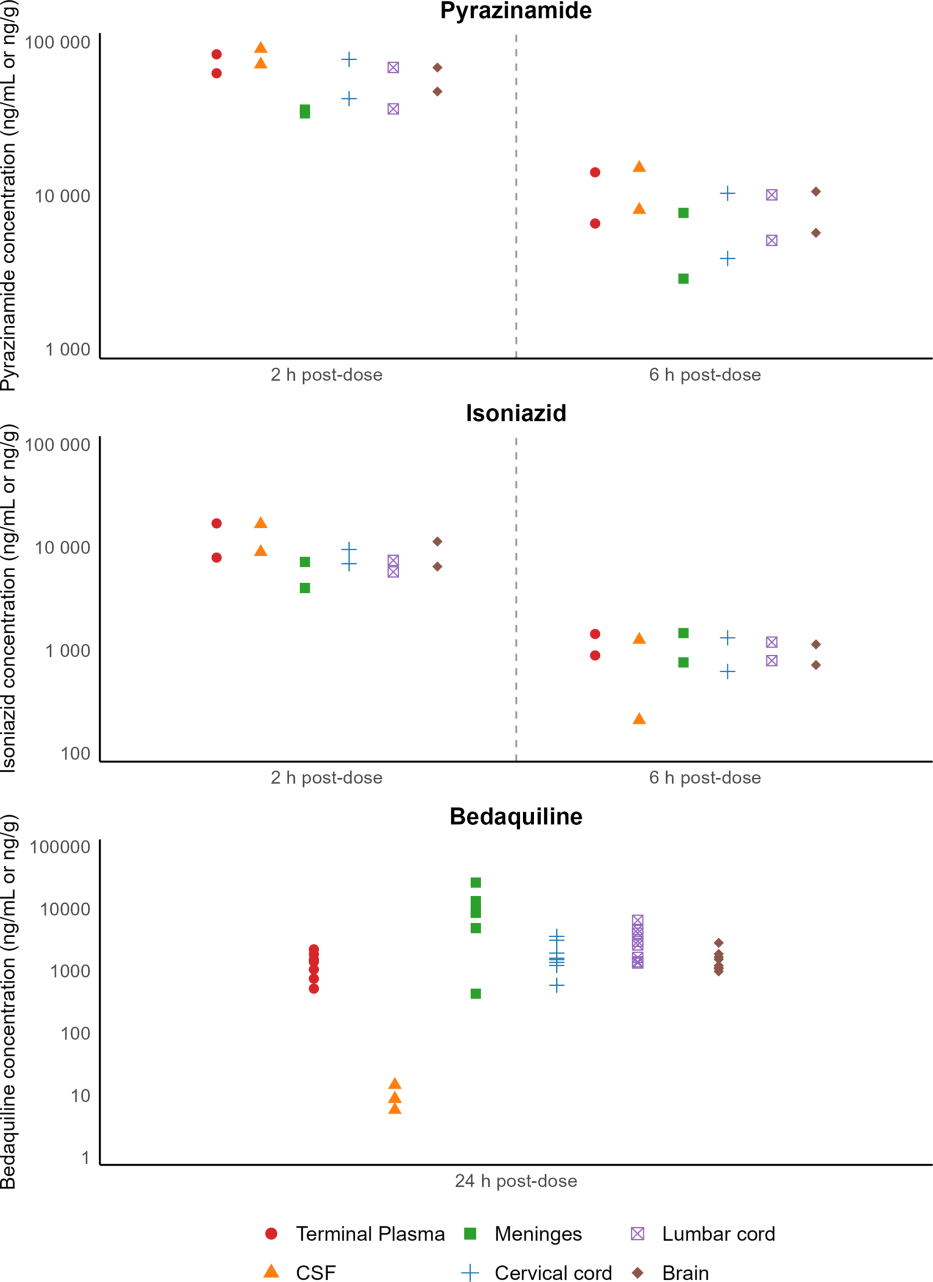
Concentrations of pyrazinamide, isoniazid, and bedaquiline in terminal plasma, CSF, and CNS tissues of rabbits with TBM. Samples were collected during necropsy at predefined post-dose timepoints, with all matrices from each animal collected at the same timepoint.

**Fig 3:**
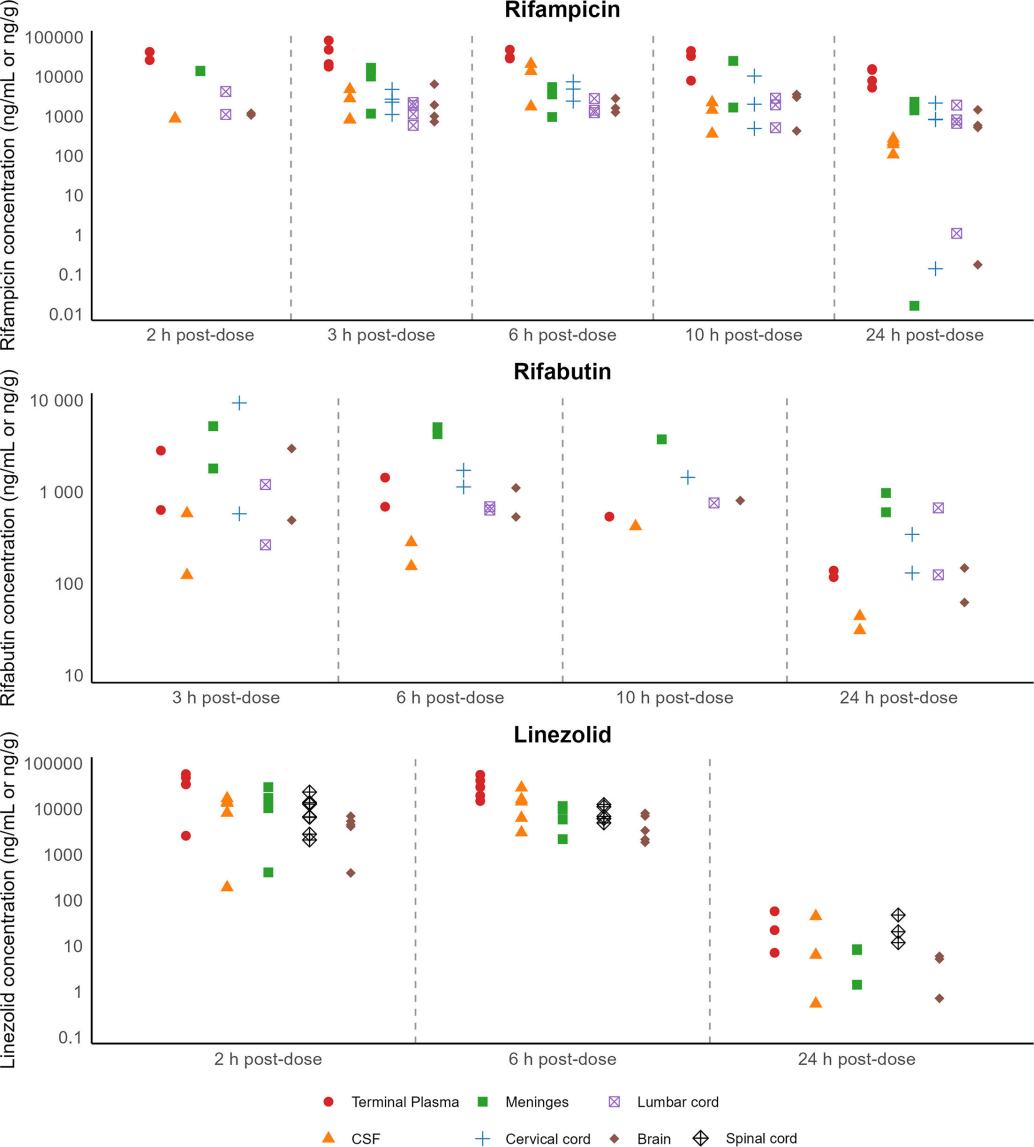
Concentrations of rifampicin, rifabutin, and linezolid in terminal plasma, CSF, and CNS tissues of rabbits with TBM. Samples were collected during necropsy at predefined post-dose timepoints, with all matrices from each animal collected at the same timepoint.

All terminal plasma, CSF, and CNS tissue samples were quantifiable for all drugs. Four bedaquiline CSF samples were excluded due to blood contamination. In the linezolid experiment, cervical and lumbar spinal cord regions were not sampled separately; instead, composite spinal cord samples were analyzed. Paired terminal concentrations of pyrazinamide, isoniazid, and bedaquiline are presented in Fig. 2, and those for rifampicin, rifabutin, and linezolid are presented in Fig. 3. Concentration-time data for the study drugs in extra-CNS tissues (lung, liver, and spleen) are presented in the (Fig. S1 and S2).

### Pharmacokinetic modeling

Plasma concentration–time data were described using first-order absorption and elimination models. Pyrazinamide was best characterized by a one-compartment model; isoniazid and bedaquiline by two-compartment models (with lag-time–delayed absorption for bedaquiline); rifampicin by a one-compartment model with absorption delayed via transit compartments; rifabutin by a two-compartment model with lag-time–delayed absorption; and linezolid by a one-compartment model with lag-time–delayed absorption. All models incorporated allometric scaling of disposition parameters based on WT. Parameter estimates with 95% confidence intervals and goodness-of-fit plots for the final plasma models, as well as model-derived plasma exposure metrics (AUC_0–24h_ and C_max_), are presented in Fig. S1 through S8 and Table S2.

**Fig 4:**
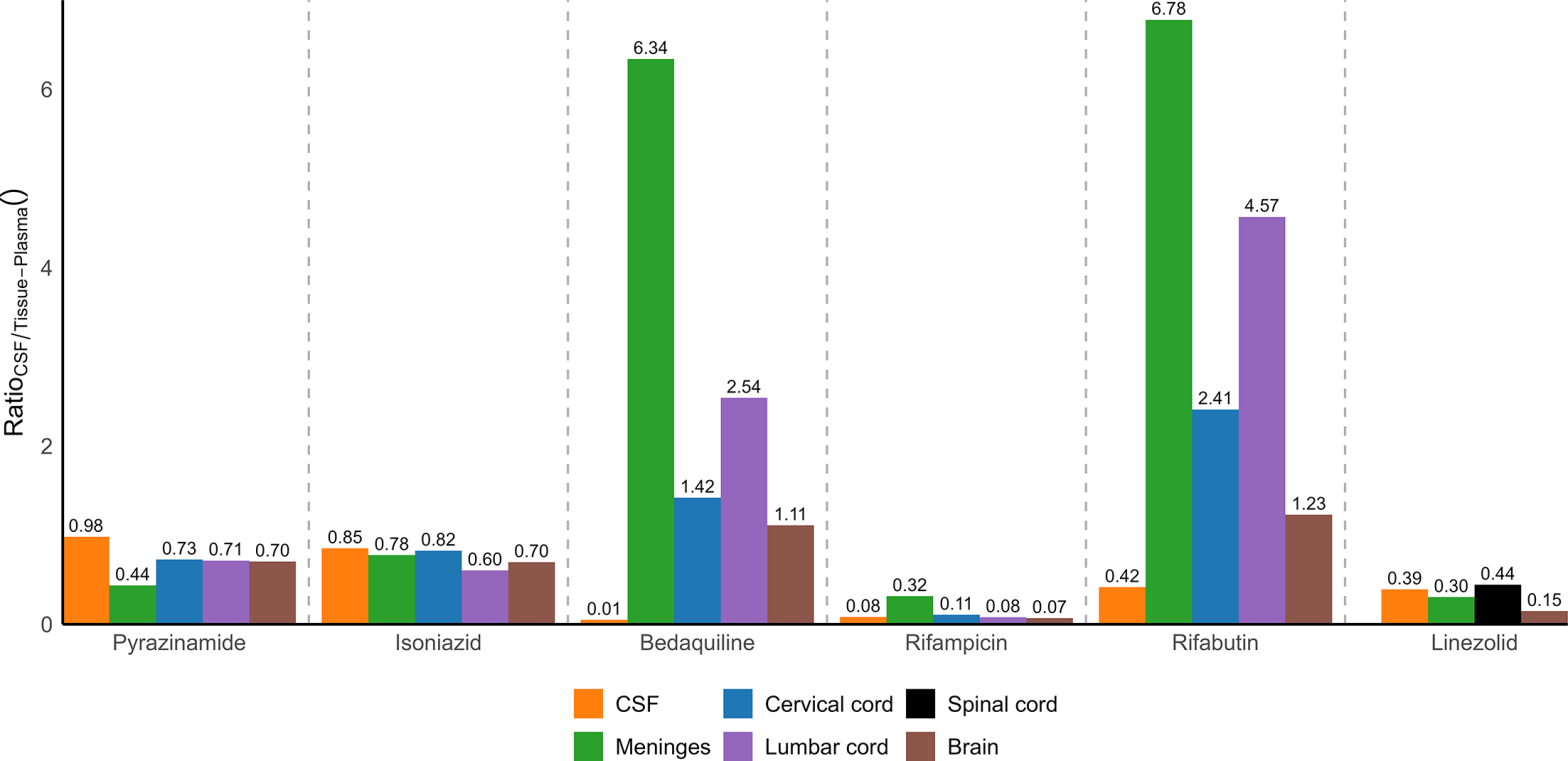
CSF and CNS tissue penetration ratios for pyrazinamide, isoniazid, bedaquiline, rifampicin, rifabutin, and linezolid in rabbits with TBM.

All CSF and CNS tissue data were linked to the corresponding plasma models. Pyrazinamide and isoniazid penetrated well into CSF (Ratio_CSF–Plasma_ 0.98 and 0.85, respectively) and showed favorable distribution into CNS tissues (Ratio_tissue–Plasma_ ranging from 0.44 to 0.73 for pyrazinamide and 0.60 to 0.82 for isoniazid). In contrast, bedaquiline showed marked compartmental differences, with very low CSF penetration (Ratio_CSF–Plasma_ = 0.01) but substantially higher accumulation in CNS tissues (Ratio_tissue–Plasma_ ranging from 1.11 to 6.34). Rifampicin had the lowest overall CNS penetration (Ratio_CSF/tissue–Plasma_ = 0.08 in CSF and 0.07 to 0.32 in CNS tissues), whereas rifabutin penetrated more extensively in both CSF (Ratio_CSF–Plasma_ = 0.42) and CNS tissues (Ratio_tissue–Plasma_ ranging from 1.23 to 6.78). Linezolid exhibited modest penetration that was broadly consistent across compartments (Ratio_CSF/tissue–Plasma_ = 0.39 in CSF and 0.15 to 0.44 in CNS tissues). Ratio_CSF/tissue–Plasma_ values for all study drugs are presented in Fig. 4.

Tables S3 and S4 include all estimated Ratio_CSF/tissue–Plasma_ and T½_Plasma–CSF/tissue_ values, with their respective 95% confidence intervals, including estimates for extra-CNS tissues (spleen and lung).

### Simulations

**Fig 5:**
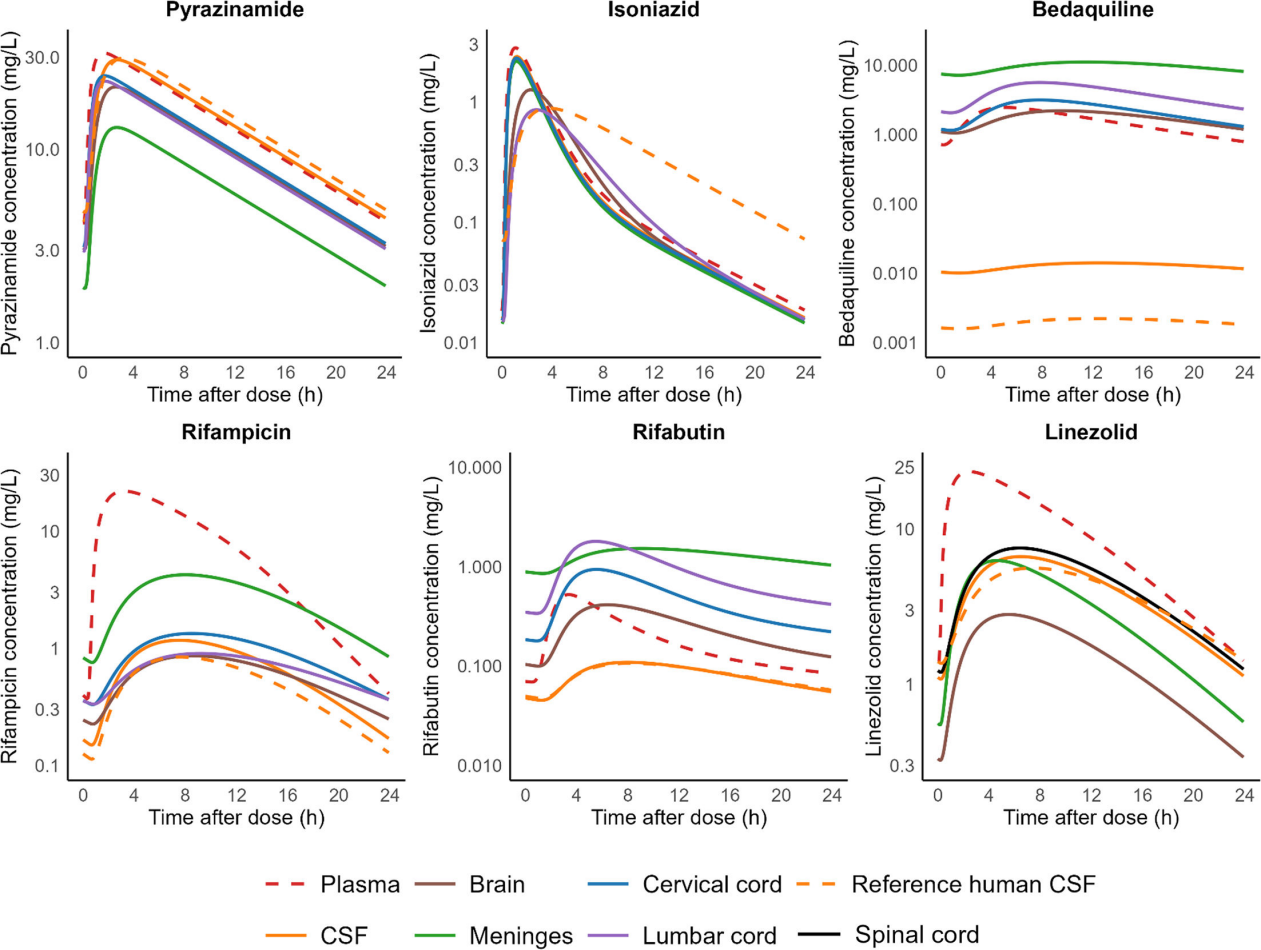
Simulated concentration–time profiles of pyrazinamide, isoniazid, bedaquiline, rifampicin, rifabutin, and linezolid in plasma, CSF, and CNS tissues of a typical TB patient (male, 55 kg). Dashed lines represent simulations informed by previously published pharmacokinetic models based on human data, whereas solid lines represent interspecies-scaled profiles extrapolated from rabbit experiments.

Simulated concentration–time profiles of the study drugs in human CSF and CNS tissues for the reference individual (WT: 55 kg) are presented in Fig. 5. CSF profiles from the reference human data and the rabbit model-derived predictions overlapped for all drugs except isoniazid due to differences in the equilibrium delay, and bedaquiline, for which the Ratio_CSF/tissue–Plasma_ was higher in the rabbit data compared with previously reported human data (0.01 vs 0.001).

## DISCUSSION

We characterized site of disease penetration of six antituberculosis drugs at human-equivalent doses using a rabbit model of TBM. Notably, the CSF penetration ratios we observed in rabbits for all study drugs closely mirrored values previously reported in clinical studies (3, 4, 10, 11, 25), supporting the translational relevance of this preclinical TBM model. Among the study drugs, pyrazinamide, isoniazid, bedaquiline, and rifabutin exhibited extensive CNS tissue distribution, linezolid showed moderate distribution, and rifampicin displayed limited penetration. The plasma exposures observed in this study also align with findings from patients with TBM (3, 4, 11, 26, 27). This supports the fidelity and potential of the rabbit TBM model for predicting human CNS penetration, as the CNS penetration we observed is more likely to reflect true drug distribution rather than species-specific differences in drug clearance.

Limited data are available on the CNS penetration of antituberculosis drugs in TBM. Site of disease drug concentrations are typically measured in CSF obtained via lumbar puncture. However, as observed in our study, drug levels in CSF do not necessarily reflect distribution across CNS tissues, especially for lipophilic drugs, limiting its utility for predicting drug exposure in the meninges and brain parenchyma, where most disease occurs (28). Structural and functional differences between the BBB and BCSFB may contribute to differential drug penetration into CSF and brain tissue. The BBB is composed of tight endothelial junctions with high expression of active efflux transporters, while the BCSFB, located at the choroid plexus, features looser junctions and a distinct transporter profile (29). Additionally, CSF is a dynamic fluid produced cranially, flowing caudally and undergoing rapid turnover, which facilitates faster drug clearance compared to the relatively static brain interstitial fluid (29). Physicochemical properties of drugs are also critical determinants of their penetration and accumulation in specific biological compartments (29). Small, hydrophilic molecules such as pyrazinamide and isoniazid can cross membranes paracellularly (13), resulting in relatively uniform distribution across various fluids and tissues, as observed in our study. In contrast, the two most lipophilic drugs in the study—bedaquiline and rifabutin—achieved substantially lower concentrations in CSF compared to other CNS tissues. Their high lipophilicity (cLogP ~7.3 and 4.7, respectively [30]) likely facilitates dissolution and retention within the lipid-rich brain parenchyma, whereas the protein- and lipid-poor composition of CSF, combined with its rapid turnover, may limit the accumulation of these compounds in that compartment (13, 29).

For pyrazinamide and isoniazid, the extensive CSF penetration found in our rabbit model is consistent with several clinical TBM cohorts (3, 31–33). In contrast, bedaquiline CSF concentrations were extremely low in our study. Bedaquiline is highly protein bound in human plasma (>99.9%) and also binds tightly to plasma proteins in other species, including rabbits (34). Since only the unbound fraction is available for distribution, this extensive protein binding likely contributes to its low CSF concentrations. A case report of a TBM patient receiving standard-dose bedaquiline documented undetectable (<0.25 mg/L) CSF concentrations after 6 weeks of treatment (35). In a series of patients with pulmonary TB (*n* = 7), in whom BBB integrity was not compromised, the mean bedaquiline CSF penetration ratio was 0.0012 (10). This value is similar to the reported plasma unbound fraction (<0.001), suggesting that free bedaquiline equilibrates into CSF. We observed a slightly higher bedaquiline CSF penetration ratio (0.01) in our study, which may be partly explained by increased permeability and reduced efflux function due to inflammation and disruption of the CNS protective barriers during TBM (28). In contrast to CSF, total bedaquiline concentrations were higher in lipid-rich CNS tissues than in plasma, indicating that bedaquiline accumulates in brain parenchyma. This extensive distribution at the site of disease, combined with potent antituberculosis activity, makes bedaquiline an attractive candidate for clinical evaluation in TBM.

Linezolid has been associated with faster clinical recovery in TBM and is being evaluated for intensified treatment in several TBM trials (36). We found modest penetration into CSF and CNS tissues in the rabbit model, aligning with clinical data from adults with HIV-associated TBM (*n* = 30) receiving 1,200 mg/day linezolid (11). Similar CSF/serum ratios of linezolid were reported in a Georgian TBM cohort (*n* = 17) treated with 600 mg/day (37).

Multiple studies have reported limited penetration of rifampicin into CSF, with concentrations reaching less than 30% of plasma levels (38–40). Doses of ≥20 mg/kg can increase both CSF and intralesional exposures in TBM although substantial interindividual variability in these concentrations has been observed (41, 42). Despite using doses equivalent to 35 mg/kg in humans, we observed poor CSF penetration of rifampicin in the rabbit TBM model (Ratio_CSF–Plasma_ = 0.08). This is consistent with data from adults with HIV-associated TBM (*n* = 49), in whom the rifampicin CSF penetration ratio was ~0.06, even with use of higher (35 mg/kg) doses (4). Similarly, a study involving South African children with TBM (*n* = 61) reported CSF penetration ratios of approximately 0.05 in both lumbar and ventricular CSF samples. That study also demonstrated low penetration into the brain extracellular fluid, as measured by microdialysis, consistent with our observation of limited CNS tissue exposure in the rabbit model (6). Restricted CNS penetration of rifampicin can be attributed to its large molecular size (~823 g/mol), high polar surface area, and high plasma protein binding (>80%) (4, 30), which reduces the free, diffusible fraction. Furthermore, rifampicin is a substrate and strong inducer of P-gp, an efflux transporter highly expressed at the BBB and also present in choroid plexus epithelial cells, which may reduce net CNS entry (43). The role of rifampicin in TBM treatment warrants re-evaluation in the context of poor CNS penetration and suboptimal clinical outcomes with rifampicin-based regimens.

A plausible alternative is rifabutin, a rifamycin with high antituberculosis potency and equivalent efficacy to rifampicin (44). Supporting potential use in TBM, and consistent with previous studies in people with HIV and non-human primates (5, 45), we observed moderate rifabutin penetration into CSF and excellent exposures in the CNS. Rifabutin shares broadly similar physicochemical properties with rifampicin (molecular weight ~847 g/mol and plasma protein binding >80%) (30, 46). However, it is a less potent pregnane X receptor inducer, and *in vitro* evidence indicates that it inhibits P-gp, which provide a plausible explanation for the extensive CNS penetration observed (47, 48).

Estimating partition coefficients from the rabbit TBM model, which generates representative clinical CSF penetration values, enabled simulation of CNS drug exposures in patients. Despite relatively low exposures, rifampicin (35 mg/kg) and linezolid (1,200 mg) are expected to achieve site of disease concentrations exceeding their respective critical concentrations (49). The concentration-time profiles for isoniazid and pyrazinamide in CNS compartments mirrored plasma, suggesting adequate exposures at standard dosing. Our simulations indicated that rifabutin and bedaquiline concentrations exceeded the critical concentration in CNS tissues, but not CSF (50). These findings should be interpreted with caution, given that pharmacokinetic efficacy targets for the study drugs have not been established for TBM, and the relative free fraction—the active form—of these drugs in the CNS is unknown.

A general limitation of preclinical studies is their incomplete translatability to patients. Species-related differences in brain tissue composition, protein binding, and BBB transporter expression or activity may affect extrapolation of rabbit CNS drug distribution to humans (51). However, we showed plasma exposures and CSF penetration ratios were similar to humans for all evaluated drugs at human-equivalent doses, supporting the predictive ability of our rabbit TBM model. Such models remain essential in TBM research, as CSF drug concentrations, although measurable in humans, correlate poorly with concentrations in brain tissue, where the bulk of pathology manifests. Our analysis relied on total drug concentrations, without accounting for the unbound, pharmacologically active fraction, potentially causing overestimation of effective exposure for highly protein-bound drugs. No companion mycobacteriology assessment was conducted, limiting evaluation of drug effect and exposure–response relationships in each tissue. In addition, the CNS tissue data were sparse, limiting our ability to fully characterize temporal delays in concentration equilibration across compartments. However, the use of nonlinear mixed-effects modeling mitigated this limitation and enabled us to obtain robust parameter estimates.

In conclusion, we show that CSF concentrations do not necessarily reflect drug distribution across deeper CNS tissues, limiting predictive value for the site of disease exposures. The observed CSF penetration ratios and plasma exposures at human-equivalent doses closely aligned with those reported in TB patients, demonstrating the translational potential of our preclinical model. The rabbit TBM model, therefore, serves as a platform for predicting drug distribution in human CNS tissues and for evaluating new drug candidates for TBM. High relative CNS exposures of bedaquiline and rifabutin support clinical evaluation for TBM therapy.

## Data Availability

The data sets supporting the findings of this study are available from the corresponding authors upon reasonable request.

## SUPPLEMENTAL MATERIAL

Supplemental material aac.00261-26-s0001.pdf Fig. S1 to S8; Tables S1 to S4.

ASM does not own the copyrights to Supplemental Material that may be linked to, or accessed through, an article. The authors have granted ASM a non-exclusive, world-wide license to publish the Supplemental Material files. Please contact the corresponding author directly for reuse.

## References

[1] Wasserman S, Davis A, Wilkinson RJ, Meintjes G. 2019. Key considerations in the pharmacotherapy of tuberculous meningitis. Expert Opin Pharmacother 20:1791–1795. doi:10.1080/14656566.2019.1638912 31305179 PMC6763362

[2] Maranchick NF, Alshaer MH, Smith AGC, Avaliani T, Gujabidze M, Bakuradze T, Sabanadze S, Avaliani Z, Kipiani M, Peloquin CA, et al.. 2022. Cerebrospinal fluid concentrations of fluoroquinolones and carbapenems in tuberculosis meningitis. Front Pharmacol 13:1048653. doi:10.3389/fphar.2022.1048653 36578553 PMC9791083

[3] Calderin JM, Wasserman S, Resendiz-Galvan JE, Abdelgawad N, Davis A, Stek C, Wiesner L, Wilkinson RJ, Denti P. 2025. Population pharmacokinetics of pyrazinamide and isoniazid in plasma and cerebrospinal fluid from South African adults with tuberculous meningitis. Antimicrob Agents Chemother 69:e0009925. doi:10.1128/aac.00099-25 40590723 PMC12327010

[4] Abdelgawad N, Wasserman S, Gausi K, Davis A, Stek C, Wiesner L, Meintjes G, Wilkinson RJ, Denti P. 2025. Population pharmacokinetics of rifampicin in plasma and cerebrospinal fluid in adults with tuberculosis meningitis. J Infect Dis 232:e234–e241. doi:10.1093/infdis/jiaf178 40295169 PMC12349950

[5] Siegal FP, Eilbott D, Burger H, Gehan K, Davidson B, Kaell AT, Weiser B. 1990. Dose-limiting toxicity of rifabutin in AIDS-related complex. AIDS 4:433–442. doi:10.1097/00002030-199005000-00009 2164820

[6] Abdelgawad N, Tshavhungwe MP, Rohlwink U, McIlleron H, Abdelwahab MT, Wiesner L, Castel S, Steele C, Enslin JN, Thango NS, et al.. 2023. Population pharmacokinetic analysis of rifampicin in plasma, cerebrospinal fluid, and brain extracellular fluid in South African children with tuberculous meningitis. Antimicrob Agents Chemother 67:e0147422. doi:10.1128/aac.01474-22 36815838 PMC10019224

[7] Liu L, Collier AC, Link JM, Domino KB, Mankoff DA, Eary JF, Spiekerman CF, Hsiao P, Deo AK, Unadkat JD. 2015. Modulation of p-glycoprotein at the human blood-brain barrier by quinidine or rifampin treatment: a positron emission tomography imaging study. Drug Metab Dispos 43:1795–1804. doi:10.1124/dmd.114.058685 26354948 PMC4613948

[8] Grassi C, Peona V. 1996. Use of rifabutin in the treatment of pulmonary tuberculosis. Clin Infect Dis 22 Suppl 1:S50–4. doi:10.1093/clinids/22.supplement_1.s50 8785257

[9] Abdelwahab MT, Wasserman S, Brust JCM, Dheda K, Wiesner L, Gandhi NR, Warren RM, Sirgel FA, Meintjes G, Maartens G, et al.. 2021. Linezolid population pharmacokinetics in South African adults with drug-resistant tuberculosis. Antimicrob Agents Chemother 65:e0138121. doi:10.1128/AAC.01381-21 34543098 PMC8597769

[10] Upton CM, Steele CI, Maartens G, Diacon AH, Wiesner L, Dooley KE. 2022. Pharmacokinetics of bedaquiline in cerebrospinal fluid (CSF) in patients with pulmonary tuberculosis (TB). J Antimicrob Chemother 77:1720–1724. doi:10.1093/jac/dkac067 35257182 PMC9633714

[11] Abdelgawad N, Wasserman S, Abdelwahab MT, Davis A, Stek C, Wiesner L, Black J, Meintjes G, Wilkinson RJ, Denti P. 2024. Linezolid population pharmacokinetic model in plasma and cerebrospinal fluid among patients with tuberculosis meningitis. J Infect Dis 229:1200–1208. doi:10.1093/infdis/jiad413 37740554 PMC11011161

[12] Rizk ML, Zou L, Savic RM, Dooley KE. 2017. Importance of drug pharmacokinetics at the site of action. Clin Transl Sci 10:133–142. doi:10.1111/cts.12448 28160433 PMC5421734

[13] Nau R, Sörgel F, Eiffert H. 2010. Penetration of drugs through the blood-cerebrospinal fluid/blood-brain barrier for treatment of central nervous system infections. Clin Microbiol Rev 23:858–883. doi:10.1128/CMR.00007-10 20930076 PMC2952976

[14] Wasserman S, Antilus-Sainte R, Abdelgawad N, Odjourian NM, Cristaldo M, Dougher M, Kaya F, Zimmerman M, Denti P, Gengenbacher M. 2024. Rifabutin central nervous system concentrations in a rabbit model of tuberculous meningitis. Antimicrob Agents Chemother 68:e0078324. doi:10.1128/aac.00783-24 39028192 PMC11304741

[15] Lanni F, Antilus Sainte R, Hansen M Jr, Parigi P, Kaya F, LoMauro K, Siow B, Wilkinson RJ, Wasserman S, Podell BK, et al.. 2023. A preclinical model of tb meningitis to determine drug penetration and activity at the sites of disease. Antimicrob Agents Chemother 67:e0067123. doi:10.1128/aac.00671-23 37966227 PMC10720511

[16] Tsenova L, Ellison E, Harbacheuski R, Moreira AL, Kurepina N, Reed MB, Mathema B, Barry CE 3rd, Kaplan G. 2005. Virulence of selected Mycobacterium tuberculosis clinical isolates in the rabbit model of meningitis is dependent on phenolic glycolipid produced by the bacilli. J Infect Dis 192:98–106. doi:10.1086/430614 15942899

[17] Mould DR, Upton RN. 2013. Basic concepts in population modeling, simulation, and model-based drug development-part 2: introduction to pharmacokinetic modeling methods. CPT Pharmacometrics Syst Pharmacol 2:e38. doi:10.1038/psp.2013.14 23887688 PMC3636497

[18] Lacroix BD, Friberg LE, Karlsson MO. 2012. Evaluation of IPPSE, an alternative method for sequential population PKPD analysis. J Pharmacokinet Pharmacodyn 39:177–193. doi:10.1007/s10928-012-9240-x 22270323

[19] Sheiner LB, Stanski DR, Vozeh S, Miller RD, Ham J. 1979. Simultaneous modeling of pharmacokinetics and pharmacodynamics: application to d-tubocurarine. Clin Pharmacol Ther 25:358–371. doi:10.1002/cpt1979253358 761446

[20] Dosne A-G, Bergstrand M, Karlsson MO. 2017. An automated sampling importance resampling procedure for estimating parameter uncertainty. J Pharmacokinet Pharmacodyn 44:509–520. doi:10.1007/s10928-017-9542-0 28887735 PMC5686280

[21] Bernabe-Ortiz A, Carcamo CP, Sanchez JF, Rios J. 2011. Weight variation over time and its association with tuberculosis treatment outcome: a longitudinal analysis. PLoS One 6:e18474. doi:10.1371/journal.pone.0018474 21494617 PMC3072983

[22] Svensson E, Dosne A, Karlsson M. 2016. Population pharmacokinetics of bedaquiline and metabolite M2 in patients with drug-resistant tuberculosis: the effect of time-varying weight and albumin. CPT Pharmacometrics Syst Pharmacol 5:682–691. doi:10.1002/psp4.12147 27863179 PMC5192973

[23] Hennig S, Svensson EM, Niebecker R, Fourie PB, Weiner MH, Bonora S, Peloquin CA, Gallicano K, Flexner C, Pym A, et al.. 2016. Population pharmacokinetic drug-drug interaction pooled analysis of existing data for rifabutin and HIV pIs. J Antimicrob Chemother 71:1330–1340. doi:10.1093/jac/dkv470 26832753 PMC4830412

[24] Boxenbaum H. 1982. Interspecies scaling, allometry, physiological time, and the ground plan of pharmacokinetics. J Pharmacokinet Biopharm 10:201–227. doi:10.1007/BF01062336 7120049

[25] Garey K, Killian A, Narang P, Luer M, Dujovny M, Danziger L, Rodvold K. 1999. Penetration of rifabutin (R) into cerebrospinal fluid (CSF) of ventriculostomy patients. Clinical Pharmacology & Therapeutics 65:161–161. doi:10.1016/S0009-9236(99)80175-1

[26] Shao G, Bao Z, Davies Forsman L, Paues J, Werngren J, Niward K, Schön T, Bruchfeld J, Alffenaar J-W, Hu Y. 2023. Population pharmacokinetics and model-based dosing evaluation of bedaquiline in multidrug-resistant tuberculosis patients. Front Pharmacol 14:1022090. doi:10.3389/fphar.2023.1022090 37050904 PMC10083270

[27] Rawizza HE, Oladokun R, Ejeliogu E, Oguche S, Ogunbosi BO, Agbaji O, Odaibo G, Imade G, Olaleye D, Wiesner L, et al.. 2021. Rifabutin pharmacokinetics and safety among TB/HIV-coinfected children receiving lopinavir/ritonavir-containing second-line art. J Antimicrob Chemother 76:710–717. doi:10.1093/jac/dkaa512 33294914 PMC7879135

[28] Davis AG, Rohlwink UK, Proust A, Figaji AA, Wilkinson RJ. 2019. The pathogenesis of tuberculous meningitis. J Leukoc Biol 105:267–280. doi:10.1002/JLB.MR0318-102R 30645042 PMC6355360

[29] Johanson CE, Duncan JA, Klinge PM, Brinker T, Stopa EG, Silverberg GD. 2008. Multiplicity of cerebrospinal fluid functions: new challenges in health and disease. Cerebrospinal Fluid Res 5:10. doi:10.1186/1743-8454-5-10 18479516 PMC2412840

[30] Lakshminarayana SB, Huat TB, Ho PC, Manjunatha UH, Dartois V, Dick T, Rao SPS. 2015. Comprehensive physicochemical, pharmacokinetic and activity profiling of anti-tb agents. J Antimicrob Chemother 70:857–867. doi:10.1093/jac/dku457 25587994 PMC7714050

[31] Phuapradit P, Supmonchai K, Kaojarern S, Mokkhavesa C. 1990. The blood/cerebrospinal fluid partitioning of pyrazinamide: a study during the course of treatment of tuberculous meningitis. J Neurol Neurosurg Psychiatry 53:81–82. doi:10.1136/jnnp.53.1.81 2303836 PMC1014104

[32] Ding J, Thuy Thuong Thuong N, Pham TV, Heemskerk D, Pouplin T, Tran CTH, Nguyen MTH, Nguyen PH, Phan LP, Nguyen CVV, et al.. 2020. Pharmacokinetics and pharmacodynamics of intensive antituberculosis treatment of tuberculous meningitis. Clin Pharmacol Ther 107:1023–1033. doi:10.1002/cpt.1783 31956998 PMC7158205

[33] Kaojarern S, Supmonchai K, Phuapradit P, Mokkhavesa C, Krittiyanunt S. 1991. Effect of steroids on cerebrospinal fluid penetration of antituberculous drugs in tuberculous meningitis. Clin Pharmacol Ther 49:6–12. doi:10.1038/clpt.1991.2 1988241

[34] van Heeswijk RPG, Dannemann B, Hoetelmans RMW. 2014. Bedaquiline: a review of human pharmacokinetics and drug-drug interactions. J Antimicrob Chemother 69:2310–2318. doi:10.1093/jac/dku171 24860154

[35] Akkerman OW, Odish OFF, Bolhuis MS, de Lange WCM, Kremer HPH, Luijckx G-J, van der Werf TS, Alffenaar J-W. 2016. Pharmacokinetics of bedaquiline in cerebrospinal fluid and serum in multidrug-resistant tuberculous meningitis. Clin Infect Dis 62:523–524. doi:10.1093/cid/civ921 26534926

[36] Fei Z-T, Huang W, Zhou D-P, Yang Y, Liu P, Gan N, He P-P, Ye D, Liu H-R, Liu X-H, et al.. 2025. Clinical efficacy of linezolid in the treatment of tuberculous meningitis: a retrospective analysis and literature review. BMC Infect Dis 25:467. doi:10.1186/s12879-025-10874-x 40188033 PMC11972499

[37] Kempker RR, Smith AGC, Avaliani T, Gujabidze M, Bakuradze T, Sabanadze S, Avaliani Z, Collins JM, Blumberg HM, Alshaer MH, et al.. 2022. Cycloserine and linezolid for tuberculosis meningitis: pharmacokinetic evidence of potential usefulness. Clin Infect Dis 75:682–689. doi:10.1093/cid/ciab992 34849645 PMC9464073

[38] Donald PR. 2010. Cerebrospinal fluid concentrations of antituberculosis agents in adults and children. Tuberculosis (Edinb) 90:279–292. doi:10.1016/j.tube.2010.07.002 20709598

[39] Nau R, Prange HW, Menck S, Kolenda H, Visser K, Seydel JK. 1992. Penetration of rifampicin into the cerebrospinal fluid of adults with uninflamed meninges. J Antimicrob Chemother 29:719–724. doi:10.1093/jac/29.6.719 1506352

[40] Ellard GA, Humphries MJ, Allen BW. 1993. Cerebrospinal fluid drug concentrations and the treatment of tuberculous meningitis. Am Rev Respir Dis 148:650–655. doi:10.1164/ajrccm/148.3.650 8368635

[41] Tucker EW, Guglieri-Lopez B, Ordonez AA, Ritchie B, Klunk MH, Sharma R, Chang YS, Sanchez-Bautista J, Frey S, Lodge MA, et al.. 2018. Noninvasive ^11^c-rifampin positron emission tomography reveals drug biodistribution in tuberculous meningitis. Sci Transl Med 10:eaau0965. doi:10.1126/scitranslmed.aau0965 30518610 PMC6360528

[42] Cresswell FV, Meya DB, Kagimu E, Grint D, Te Brake L, Kasibante J, Martyn E, Rutakingirwa M, Quinn CM, Okirwoth M, et al.. 2021. High-dose oral and intravenous rifampicin for the treatment of tuberculous meningitis in predominantly human immunodeficiency virus (HIV)-positive ugandan adults: a phase II open-label randomized controlled trial. Clin Infect Dis 73:876–884. doi:10.1093/cid/ciab162 33693537 PMC8423465

[43] Miller DS, Bauer B, Hartz AMS. 2008. Modulation of p-glycoprotein at the blood-brain barrier: opportunities to improve central nervous system pharmacotherapy. Pharmacol Rev 60:196–209. doi:10.1124/pr.107.07109 18560012 PMC2634288

[44] Schmidt H, Zysk G, Reinert RR, Brück W, Stringaris A, Chen V, Stuertz K, Fischer F, Bartels R, Schaper K-J, et al.. 1997. Rifabutin for experimental pneumococcal meningitis. Chemotherapy 43:264–271. doi:10.1159/000239577 9209783

[45] Benedetti MS, Pianezzola E, Brughera M, Fraier D, Castelli MG. 1994. Concentrations of rifabutin in plasma and cerebrospinal fluid in cynomolgus monkeys. J Antimicrob Chemother 34:600–603. doi:10.1093/jac/34.4.600 7868414

[46] Blaschke TF, Skinner MH. 1996. The clinical pharmacokinetics of rifabutinclinical infectious diseases

[47] Nilles J, Weiss J, Sauter M, Haefeli WE, Ruez S, Theile D. 2023. Comprehensive in vitro analysis evaluating the variable drug-drug interaction risk of rifampicin compared to rifabutin. Arch Toxicol 97:2219–2230. doi:10.1007/s00204-023-03531-2 37285043 PMC10322781

[48] Phondeth L, Kamaraj R, Nilles J, Weiss J, Haefeli WE, Pávek P, Theile D. 2024. Rifabutin but not rifampicin can partly out-balance p-glycoprotein induction by concurrent p-glycoprotein inhibition through high affinity binding to the inhibitory site. Arch Toxicol 98:223–231. doi:10.1007/s00204-023-03618-w 37833491 PMC10761502

[49] Hajizadeh M, Hekmatpour N, Rahimlou B, Eskandari A, Zamani MS, Ferdosnejad K, Sakhaee F, Masoumi M, Marjani M, Tarashi S, et al.. 2025. Prevalence and genetic insights of linezolid resistance in tuberculosis: a study of sensitive and resistant clinical isolates. Diagn Microbiol Infect Dis 111:116719. doi:10.1016/j.diagmicrobio.2025.116719 39899947

[50] World Health Organization. 2018. Technical report on critical concentrations for drug susceptibility testing of medicines used in the treatment of drug-resistant tuberculosis. Geneva: World Health Organization; 2018 (WHO/CDS/TB/2018.5). Licence: CC BY-NC-SA 3.0 IGO

[51] Syvänen S, Lindhe O, Palner M, Kornum BR, Rahman O, Långström B, Knudsen GM, Hammarlund-Udenaes M. 2009. Species differences in blood-brain barrier transport of three positron emission tomography radioligands with emphasis on p-glycoprotein transport. Drug Metab Dispos 37:635–643. doi:10.1124/dmd.108.024745 19047468

