# Supplementary material for: Central nervous system antituberculosis drug exposures in a rabbit model of tuberculous meningitis
Source: Antimicrob Agents Chemother. 2026 Jul 10;70(8):e00261-26. doi: 10.1128/aac.00261-26 (PMC13436124; doi:10.1128/aac.00261-26)
Supplement: Supplementary file 1 [file aac.00261-26-s0001.pdf]

Central nervous system antituberculosis drug exposures in a rabbit model of tuberculous meningitis

Jose M Calderin[1], Sean Wasserman[2,3], Rosleine Antilus-Sainte[4], Melissa Cristaldo[4], Narineh M Odjourian[4], Faye Lanni[4], Firat Kaya[4], Noha Abdelgawad[1], Juan Eduardo Resendiz-Galvan[1], Matthew Zimmerman[4], Veronique Dartois[4,5], Paolo Denti[1#], Martin Gengenbacher[4,5#]

1. Division of Clinical Pharmacology, Department of Medicine, University of Cape Town, South Africa
2. Wellcome Discovery Research Platforms in Infection, Centre for Infectious Diseases Research in Africa, Institute of Infectious Disease and Molecular Medicine, University of Cape Town, Observatory, Cape Town, South Africa
3. Blizard Institute, Queen Mary University of London, London, United Kingdom
4. Center for Discovery and Innovation, Hackensack Meridian Health, Nutley, New Jersey, United States
5. Hackensack Meridian School of Medicine, Nutley, New Jersey, United States

Running head: TB Drug Exposure in the CNS

[#]Shared correspondence (by alphabetical order)

Paolo Denti
paolo.denti@uct.ac.za
Martin Gengenbacher
martin.gengenbacher@gmail.com

S1. FORMULATION OF THE STUDY DRUGS

Pyrazinamide/isoniazid, rifampicin, rifabutin and linezolid were formulated in 0.5% carboxymethyl cellulose/0.5% Tween 80/sterile water. Bedaquiline was formulated in 20% hydroxypropyl-ß-cyclodextrin in 50 mM Sodium Citrate pH 3/sterile water.

S2. SAMPLE QUANTIFICATION

Pyrazinamide, isoniazid, rifampicin, bedaquiline, rifabutin and linezolid were purchased from Sigma-Aldrich. Deuterated internal standards for each analyte were obtained from Toronto Research Chemicals. Drug-free K2EDTA plasma, whole blood, cerebrospinal fluid (CSF), liver, spleen, lung, and CNS tissues from New Zealand White (NZW) rabbits were obtained in-house and used as blank matrices for preparation of calibration standards and quality control (QC) samples.

Tissue samples were collected in 1.5 mL Precellys tubes and diluted 1:4 (weight:volume) with phosphate-buffered saline (PBS). Diluted tissues were homogenized using a Fisherbrand Bead Mill 24 for 3 minutes. Neat 1 mg/mL compound stock solutions prepared in DMSO were serially diluted in 50:50 acetonitrile:water to generate standard curve and QC spiking solutions. Calibration standards and QC samples were prepared by spiking 10 µL of the appropriate working solution into 90 µL of drug-free plasma, CSF, or control tissue homogenate.

Sample extraction for standards, QCs, and study samples was performed by adding 200 µL of 1:1 acetonitrile:methanol containing internal standards (150 ng/mL pyrazinamide-d4, 100 ng/mL isoniazid-d4, 10 ng/mL bedaquiline-d8, 20 ng/mL rifampicin-d8, 20 ng/mL rifabutin-d7, and 40 ng/mL linezolid-d8) to 20 µL of plasma, CSF, or homogenized tissue. Samples were vortexed for 5 minutes and centrifuged at 4,000 rpm for 5 minutes. One hundred microliters of supernatant were transferred to a 96-well plate for LC–MS/MS injection.

For bedaquiline CSF samples, extraction was performed directly in the CSF collection tubes. Bedaquiline has non-specific binding to sampling tubes in frozen CSF and the extraction directly in the collection tube extracts BDQ off the walls of the tube. For rifampicin standards, QCs, and samples, 10 µL of 75 mg/mL ascorbic acid was added to each sample to stabilize rifampicin in the autosampler. For isoniazid standards, QCs, and samples, 100 µL of 2%

cinnamaldehyde in methanol was added, and samples were vortexed for 30 minutes to derivatize isoniazid.

LC–MS/MS analysis was performed using a Sciex QTRAP 6500+ triple-quadrupole mass spectrometer coupled to a Shimadzu Nexera X2 UHPLC system. Chromatographic separation was achieved on a Phenomenex Luna Omega C18 Polar column (2.1 × 100 mm, 3 µm particle size) using a reverse-phase gradient at a flow rate of 0.6 mL/min. The aqueous mobile phase consisted of Milli-Q water with 0.1% formic acid, and the organic mobile phase consisted of acetonitrile with 0.1% formic acid. The gradient was programmed from 2% to 90% organic phase over 3 minutes, held at 90% for 1 minute, and re-equilibrated at 2% for 1 minute, resulting in a total run time of 5 minutes.

Quantification was performed using multiple-reaction monitoring (MRM) in positive electrospray ionization mode. The monitored precursor/product ion transitions (m/z) were 124.20/81.10 and 128.20/84.10 for pyrazinamide and pyrazinamide-d4; 252.20/80.30 and 256.20/84.30 for isoniazid and isoniazid-d4; 555.00/58.00 and 561.00/64.00 for bedaquiline and bedaquiline-d8; 823.50/791.60 and 831.50/799.60 for rifampicin and rifampicin-d8; 847.60/755.60 and 854.60/762.60 for rifabutin and rifabutin-d7; and 338.00/235.00 and 346.15/304.20 for linezolid and linezolid-d8.

Sample analysis was considered acceptable if QC concentrations were within ±20% of nominal values. Data acquisition and processing were performed using Analyst software version 1.6.3 (Sciex).

S3. PHARMACOKINETIC MODELLING

Nonlinear mixed-effects modelling was conducted using NONMEM version 7.5.1 to develop separate population pharmacokinetic models describing the concentration of the study drugs in plasma, CSF and CNS tissues. The first-order conditional estimation with eta-epsilon interaction (FOCE-I) algorithm was used throughout the modelling process. Models were

developed using a sequential, non-simultaneous approach, first describing plasma concentrations, followed by the stepwise incorporation of CSF and other CNS tissue data.

To describe plasma data from each experiment, one- and two-compartment pharmacokinetic models with first-order absorption (with or without a lag time) and first-order elimination were tested. The effect of body size on disposition parameters was implemented using allometric scaling based on total body weight, with exponents fixed to 0.75 for clearance and 1 for volume of distribution. Random effects were included on pharmacokinetic parameters, assuming a log-normal distribution. Between-subject variability (BSV) was explored for disposition parameters, and between-occasion variability (BOV) for bioavailability and absorption parameters, with each occasion defined as a dosing event and its subsequent observations. Model development followed a stepwise approach, using the likelihood ratio test to compare nested models. A p-value of 0.05 was used for inclusion of an additional parameter, and 0.01 for retention. Beyond statistical significance, model development was guided by diagnostic plots, including visual predictive checks (VPCs) and individual fits.

Individual plasma pharmacokinetic parameters were fixed, and CSF and CNS tissue concentrations were described using hypothetical effect compartments, each linked to the central (plasma) compartment. This approach characterizes drug penetration into CSF and CNS tissues by estimating the plasma-to-CSF/tissue equilibration half-life ($T½_{Plasma–CSF/tissue}$), and the CSF/tissue-to-plasma ratio ($Ratio_{CSF/tissue–Plasma}$). These parameters reflect the delay in concentration equilibration between plasma and these compartments and the extent of drug exposure in CSF or CNS tissue relative to plasma at steady state.

Residual unexplained variability (RUV) was described using a combined additive and proportional error model, with the additive component constrained to be at least 20% of the assay's lower limit of quantification (LLOQ). Concentrations below the LLOQ (BLQ) were

handled using the M7+ method proposed by Wijk et al.(1), whereby BLQ values were imputed as zero, and the additive error was increased by 100% of the respective LLOQ for those observations.

The precision of the parameter estimates in the final models, indicated by 95% confidence intervals, was evaluated through the sampling importance resampling (SIR) method.

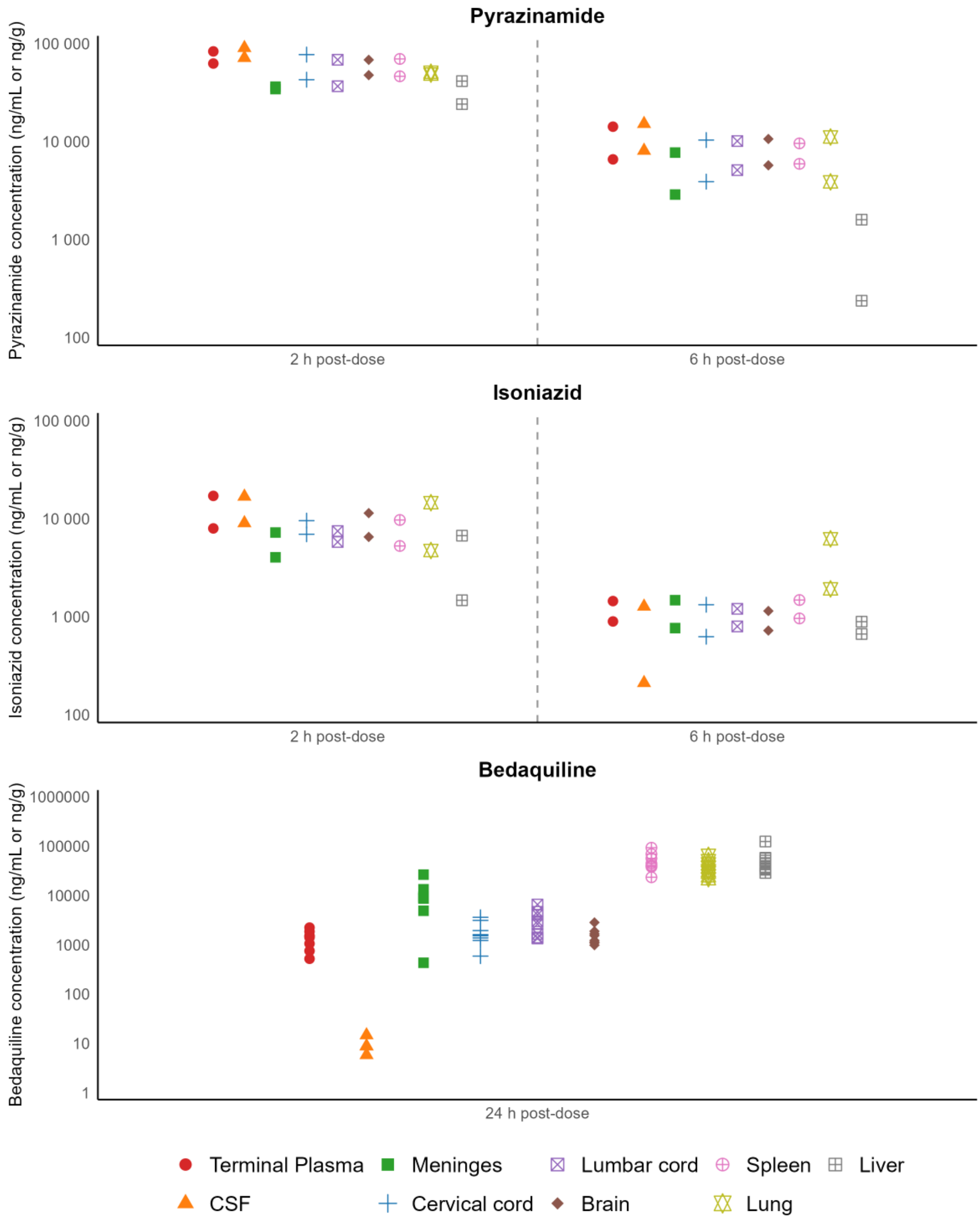

Figure S1. Concentrations of pyrazinamide, isoniazid, and bedaquiline in terminal plasma, CSF, CNS tissues, and extra-CNS tissues of rabbits with TBM. Samples were collected during necropsy at predefined post-dose timepoints, with all matrices from each animal collected at the same timepoint.

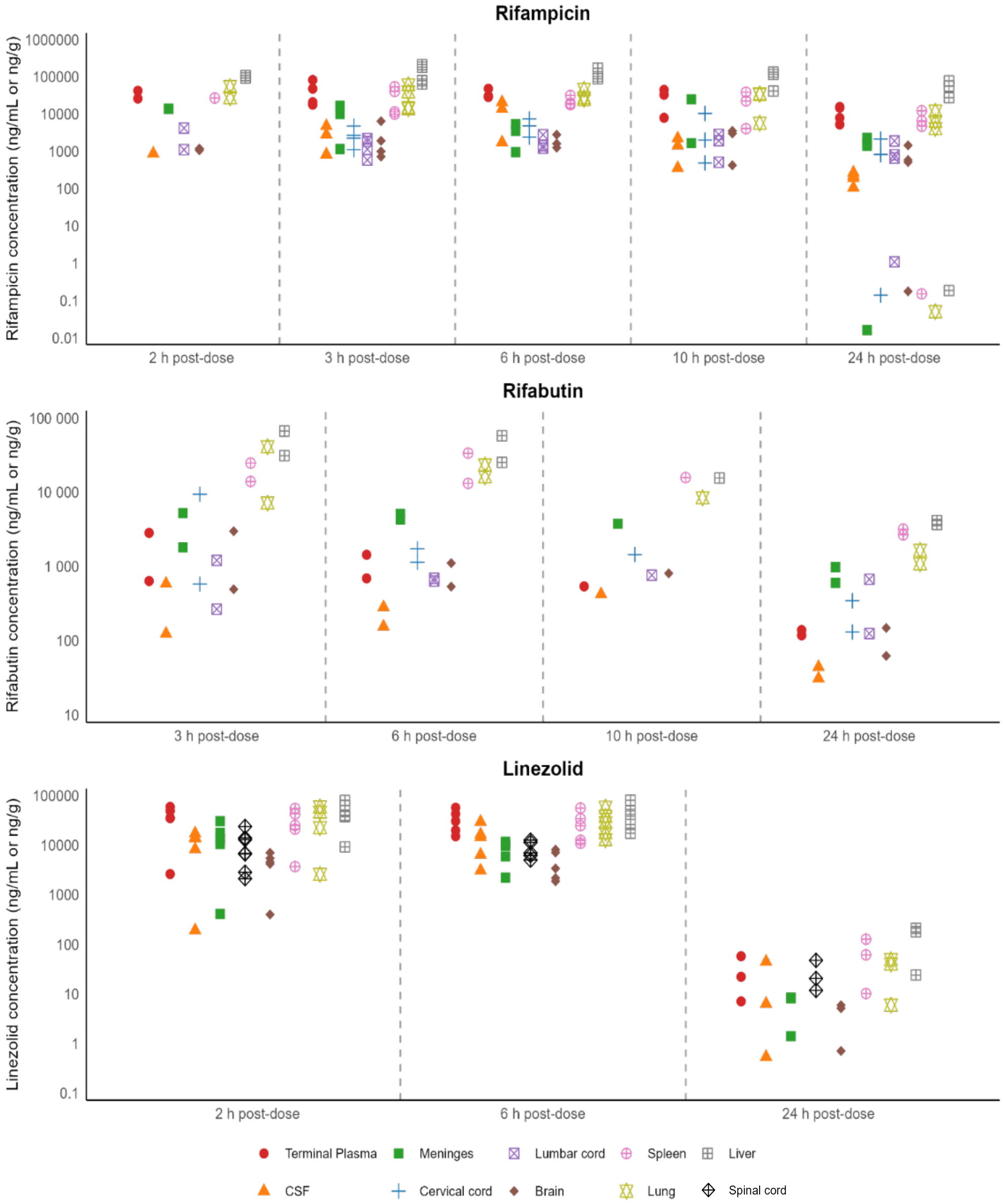

Figure S2. Concentrations of rifampicin, rifabutin, and linezolid in terminal plasma, CSF, CNS, and extra-CNS tissues of rabbits with TBM. Samples were collected during necropsy at predefined post-dose timepoints, with all matrices from each animal collected at the same timepoint.

Table S1. Final plasma pharmacokinetic parameters.

Parameter (units)	Typical values (95% CI) [a]					
	Pyrazinamide	Isoniazid	Bedaquiline	Rifampicin	Rifabutin	Linezolid
CL (L/h) [b, d]	1.71 (1.26–2.33)	2.91 (2.08–4.06)	5.92 (3.10–9.77)	0.572 (0.446–0.722)	4.82 (3.19–6.86)	1.51 (1.30–1.72)
Vc (L) [b, d]	2.94 (2.50–3.41)	6.77 (4.50–9.69)	103 (56.5–177)	5.99 (4.92–7.35)	49.6 (29.6–76.1)	3.93 (3.34–4.53)
Q (L/h) [b, d]	-	0.22 (0.11–0.39)	11.5 (5.49–18.3)	-	5.07 (2.08–8.25)	-
Vp (L) [b, d]	-	3.41 (1.37–7.30)	474 (176–763)	-	68.0 (21. 9–126)	-
F (.)	1 Fixed	1 Fixed	1 Fixed	1 Fixed	1 Fixed	1 Fixed
MTT (h)	-	-	-	0.123 (0.0617–0.215)	-	-
NN (.)	-	-	-	5.16 (2.78–7.91)	-	-
Tlag (h)	-	-	1.19 (0.763–1.50)	-	0.395 (0.324–0.493)	0.286 (0.22–0.36)
Ka (h^{-1})	1.48 (1.20–1.78)	1.75 (1.03–3.05)	0.0862 (0.048–0.155)	1.13 (0.748–1.73)	0.405 (0.216–0.705)	0.861 (0.60–1.24)
BSVCL (%)	28.1 (18.7–46.1)	7.60 (2.27–12.8)	55.6 (24.6–80.0)	26.8 (18.1–36.0)	33.9 (13.0–51.2)	10.6 (7.17–14.0)
BOVF (%)	16.3 (9.95–20.7)	57.2 (37.0–83.2)	40.6 (24.9–53.5)	42.0 (29.5–59.1)	59.9 (39.4–82.2)	25.0 (15.4–35.6)
BOVMTT (%)	-	-	-	160.9 (111–219)	-	-
BOVKa (%)	8.70 (4.83–13.8)	53.3 (28.2–74.5)	52.4 (33.0–75.8)	114.5 (82.9–147)	76.9 (49.7–108)	86.8 (64.5–116)
BOVTlag (%)	-	-	-	-	-	44.6 (26.3–58.0)
σ_prop (%)	12.4 (9.60–17.1)	25.7 (19.6–35.2)	7.60 (1.2–14.5)	27.7 (24.6–31.7)	16.5 (12.2–22.3)	40.0 (33.6–48.7)
σ_add (mg/L) [c]	0.0162 (0.00801–0.0231)	0.0244 (0.0126–0.0451)	0.168 (0.127–0.221)	0.002 Fixed	0.0247 (0.0141–0.0378)	0.0106 (0.00465–0.0180)

CL: Clearance; Vc: Central volume of distribution; Q: Intercompartmental clearance; Vp: Peripheral volume of distribution; F: Bioavailability; MTT: Mean absorption transit time; N: Number of absorption transit compartments; Tlag: Lag time; Ka: First-order absorption rate constant; BSVCL: Between-subject variability in clearance; BOVF: Between-occasion variability in bioavailability; BOVMTT: Between-occasion variability in mean absorption transit time; BOVKa: Between-occasion variability in absorption rate constant; BOVTlag: Between-occasion variability in lag time; σ_prop: Proportional error; σ_add: Additive error.

[a] Values in parentheses represent the 95% confidence interval, computed using sampling importance resampling (SIR) on the final models.

[b] All disposition parameters were allometrically scaled. Reported typical values correspond to the typical subject (median weight) in each experimental cohort.

[c] For rifampicin, the estimate of the additive component of the residual unexplained variability did not differ significantly from its lower boundary of 20% of the LLOQ and was therefore fixed to this value.

[d] Disposition parameters are apparent and estimated relative to the unknown true bioavailability.

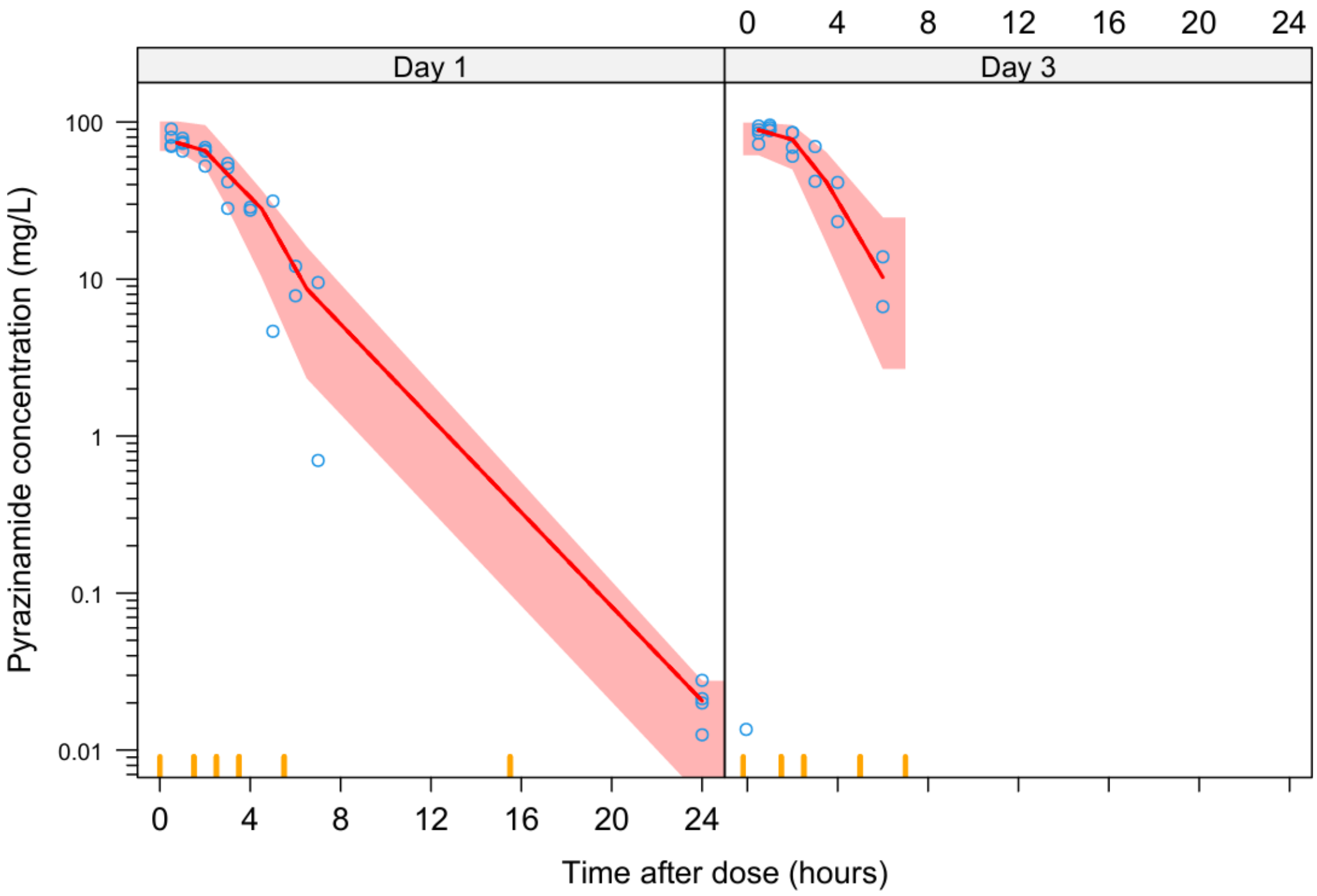

Figure S3. Visual predictive check of pyrazinamide plasma concentrations versus time after dose on day 1 and day 3 after treatment initiation. Circles represent observed data, the solid line indicates the observed 50th percentile, and the shaded area represents the model-predicted 95% confidence interval for the same percentile.

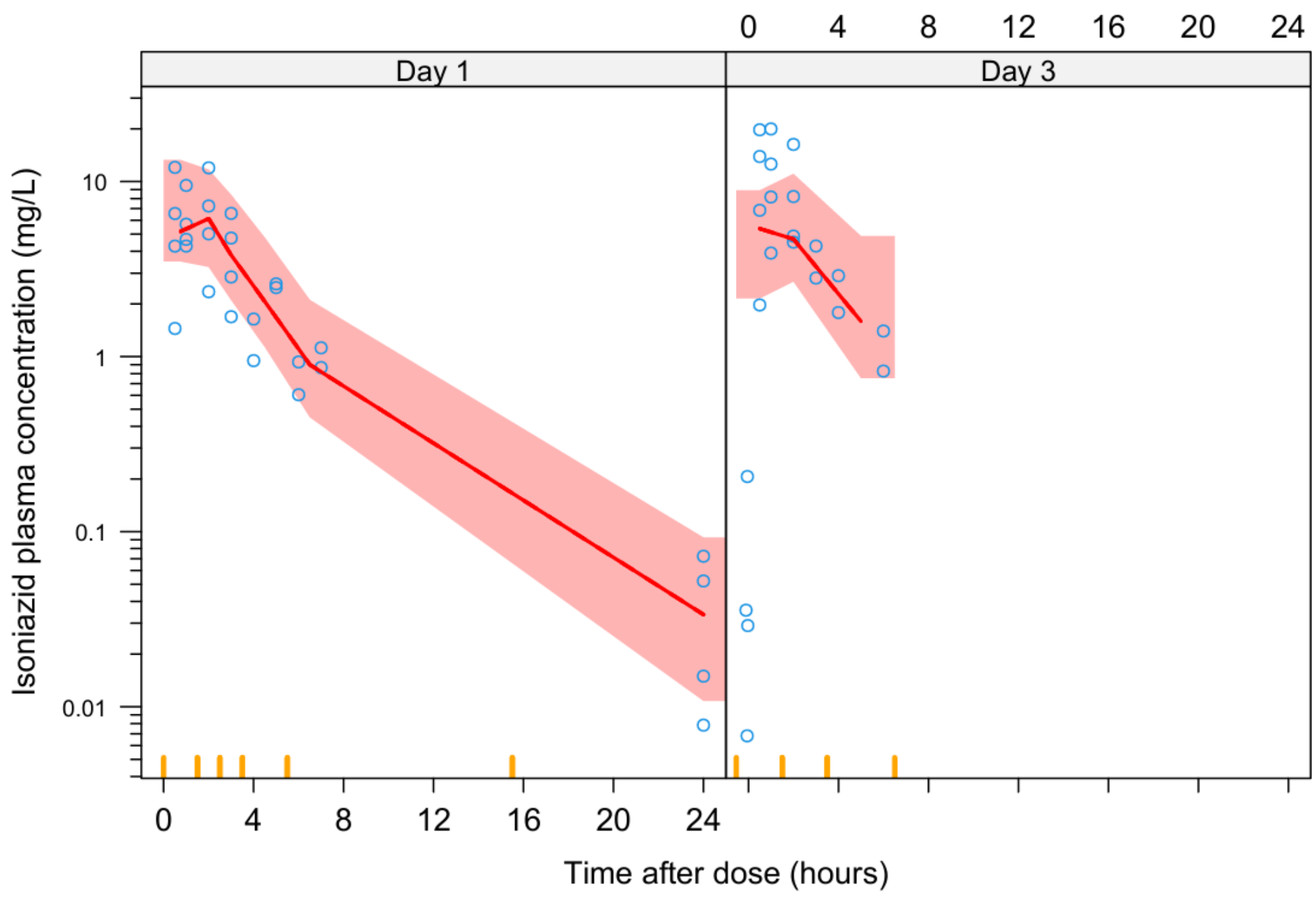

Figure S4. Visual predictive check of isoniazid plasma concentrations versus time after dose on day 1 and day 3 after treatment initiation. Circles represent observed data, the solid line indicates the observed 50th percentile, and the shaded area represents the model-predicted 95% confidence interval for the same percentile.

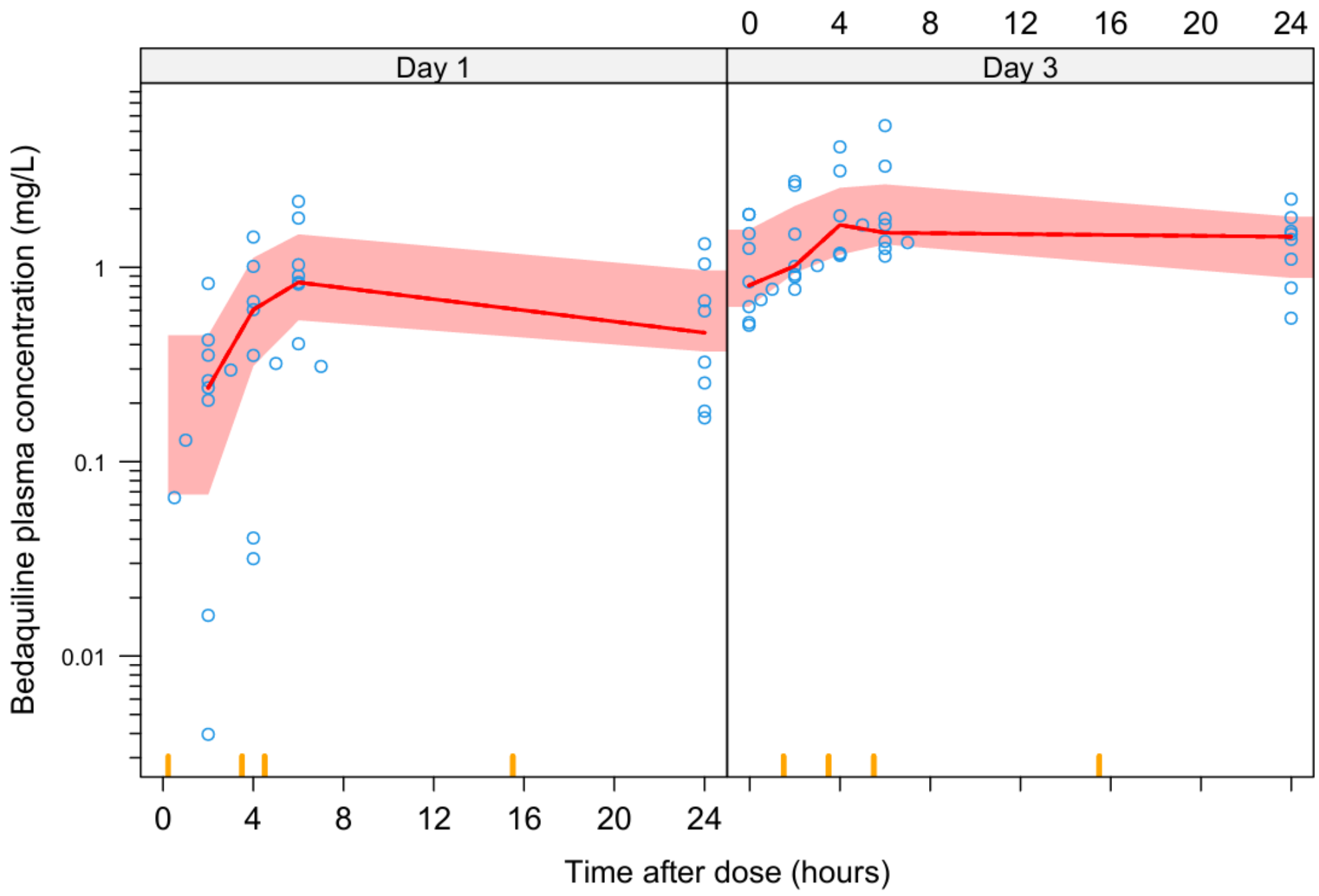

Figure S5. Visual predictive check of bedaquiline plasma concentrations versus time after dose on day 1 and day 3 after treatment initiation. Circles represent observed data, the solid line indicates the observed 50th percentile, and the shaded area represents the model-predicted 95% confidence interval for the same percentile.

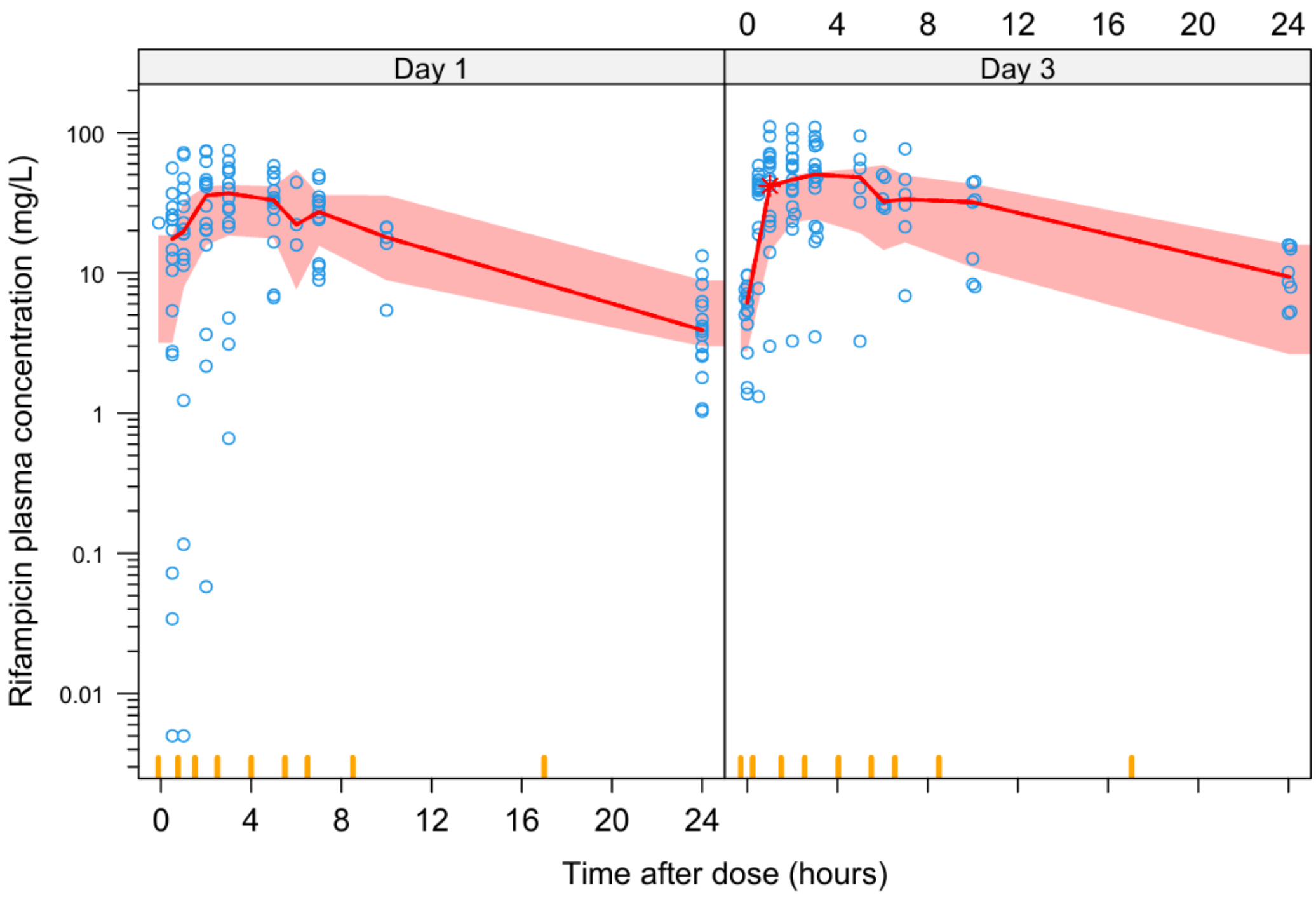

Figure S6. Visual predictive check of rifampicin plasma concentrations versus time after dose on day 1 and day 3 after treatment initiation. Circles represent observed data, the solid line indicates the observed 50th percentile, and the shaded area represents the model-predicted 95% confidence interval for the same percentile.

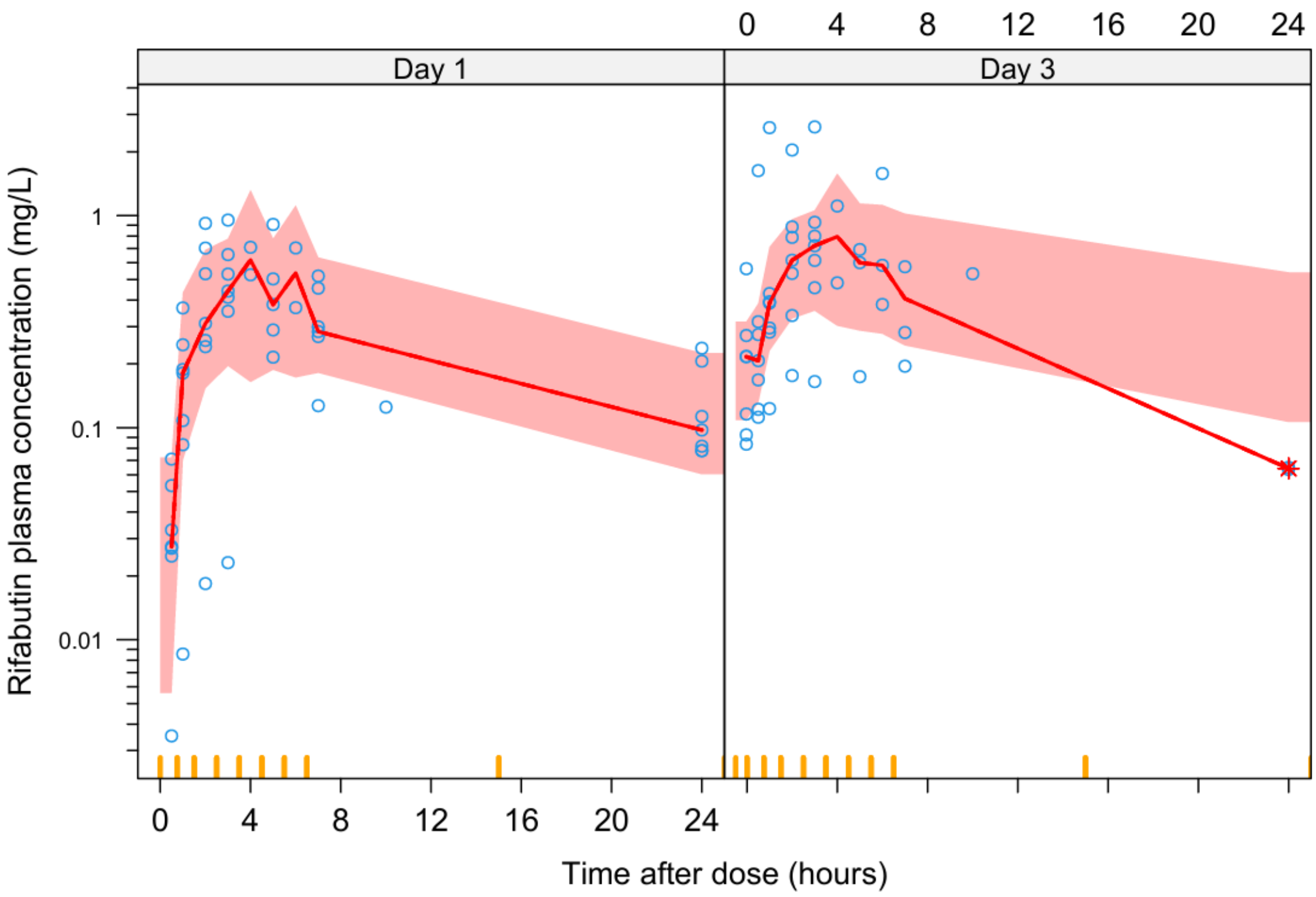

Figure S7. Visual predictive check of rifabutin plasma concentrations versus time after dose on day 1 and day 3 after treatment initiation. Circles represent observed data, the solid line indicates the observed 50th percentile, and the shaded area represents the model-predicted 95% confidence interval for the same percentile.

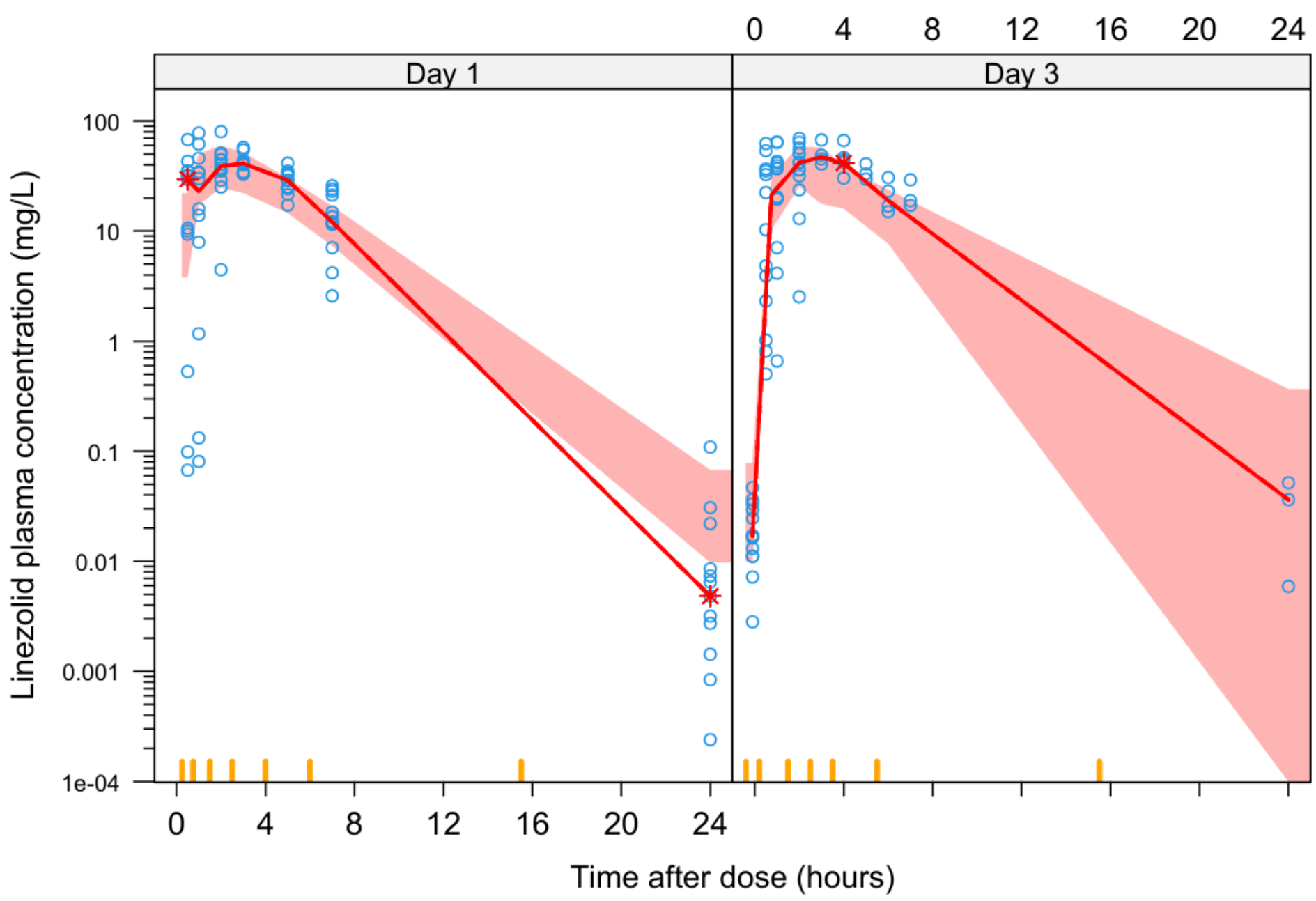

Figure S8. Visual predictive check of linezolid plasma concentrations versus time after dose on day 1 and day 3 after treatment initiation. Circles represent observed data, the solid line indicates the observed 50th percentile, and the shaded area represents the model-predicted 95% confidence interval for the same percentile.

Table S2. Model-derived plasma $AUC_{0–24h}$ and C_{max} on day 1 in TBM rabbits for each drug.

Drug	AUC_{0-24h} (mg·h/L)	C_{max} (mg/L)
Pyrazinamide	343.23 (190.77–364.37)	79.37 (70.88–90.16)
Isoniazid	26.81 (19.91–47.29)	6.47 (5.02–12.04)
Bedaquiline	13.65 (5.95–38.19)	0.96 (0.32–2.18)
Rifampicin	495.97 (161.0–766.87)	43.85 (9.80–74.8)
Rifabutin	5.40 (2.36–11.79)	0.52 (0.21–0.95)
Linezolid	318.21 (183.23–420.94)	42.0 (34.4–80.1)

Table S3. Final CSF and CNS tissue penetration pharmacokinetic parameters for pyrazinamide and isoniazid.[b]

CNS matrix	Typical values (95% CI) [a]		
	$Ratio_{CSF/tissue–Plasma}$ ()	$T½_{Plasma–CSF/tissue}$ (min)	Proportional error (%)
	**Pyrazinamide**		
CSF	0.98 (0.90–1.08)	12.5 (6.53–19.2)	6.90 (4.45–11.7)
Meninges	0.44 (0.36–0.54)	12.3 (5.95–25.9)	19.9 (12.3–30.6)
Cervical cord	0.73 (0.63–0.82)	1.68 (1.66–1.69)	13.4 (8.0–23.1)
Lumbar cord	0.71 (0.67–0.75)	1.69 (1.66–1.70)	5.42 (3.42–8.35)
Brain	0.70 (0.65–0.76)	11.3 (6.37–17.5)	11.7 (9.07–16.4)
Spleen	0.70 (0.63–0.78)	10.4 (5.26–18.6)	8.99 (5.77–14.5)
Lung	0.61 (0.50–0.75)	11.5 (6.67–25.2)	18.2 (11.1–31.5)
	**Isoniazid**		
CSF	0.85 (0.57–1.21)	1.87 (1.44–3.77)	39.9 (23.5–65.7)
Meninges	0.78 (0.52–1.09)	1.39 (1.37–1.47)	37.8 (21.6–63.5)
Cervical cord	0.82 (0.69–1.03)	1.39 (1.29–1.49)	14.7 (9.16–21.5)
Lumbar cord	0.60 (0.54–0.68)	44.1 (33.6–57.7)	8.10 (5.37–12.5)
Brain	0.70 (0.64–0.75)	25.8 (18.3–33.4)	11.3 (8.45–16.6)
Spleen	0.91 (0.65–1.29)	1.77 (1.44–3.26)	30.9 (17.6–50.9)
Lung	4.65 (3.14–7.01)	340 (192–649)	39.0 (23.4–61.8)

[a] Values in parentheses represent the 95% confidence interval, computed using sampling importance resampling (SIR) on the final models.

[b] The estimate of the additive component of the residual unexplained variability did not differ significantly from its lower boundary of 20% of the LLOQ and was therefore fixed to this value (pyrazinamide, 0.01 mg/L; isoniazid, 0.002 mg/L).

Table S4. Final CSF and CNS tissue penetration pharmacokinetic parameters for bedaquiline, rifampicin, rifabutin, and linezolid. [b]

CNS matrix	Typical values (95% CI) [a]		
	$Ratio_{CSF/tissue–Plasma}$ ()	$T½_{Plasma–CSF/tissue}$ (h)	Proportional error (%)
	**Bedaquiline**		
CSF	0.010 (0.006–0.018)	10.1 (7.56–16.8)	41.7 (32.6–58.7)
Meninges	6.34 (4.64–9.03)	4.91 (1.87–11.1)	47.2 (30.5–77.5)
Cervical cord	1.42 (1.03–1.84)	0.02 (0.0124–0.114)	39.3 (25.8–65.0)
Lumbar cord	2.54 (1.72–3.69)	0.78 (0.387–2.55)	56.2 (35.7–86.1)
Brain	1.11 (0.99–1.22)	1.55 (0.877–5.76)	25.3 (20.7–32.5)
Spleen	38.3 (28.5–46.8)	1.36 (0.725–4.02)	45.8 (29.3–75.4)
Lung	30.6 (22.2–41.1)	0.76 (0.428–2.92)	45.7 (29.7–77.6)
	**Rifampicin**		
CSF	0.08 (0.05–0.12)	1.55 (0.66–2.41)	99.5 (94.6–99.9)
Meninges	0.32 (0.21–0.45)	1.86 (0.98–2.85)	34.6 (23.8–56.1)
Cervical cord	0.11 (0.08–0.15)	2.31 (1.15–3.70)	51.2 (37.4–72.4)
Lumbar cord	0.08 (0.06–0.10)	3.41 (1.69–5.15)	42.6 (31.0–65.3)
Brain	0.07 (0.05–0.09)	2.42 (1.73–3.42)	54.7 (45.0–71.9)
Spleen	0.72 (0.61–0.88)	1.88 (1.02–2.68)	12.2 (9.0–17.5)
Lung	0.83 (0.73–0.93)	0.58 (0.22–0.90)	9.75 (7.39–14.5)
	**Rifabutin**		
CSF	0.42 (0.31–0.56)	2.97 (1.7–5.91)	33.3 (22.1–51.8)

Meninges	6.78 (5.53–8.22)	4.68 (2.8–8.76)	20.0 (13.4–31.3)
Cervical cord	2.41 (1.76–3.46)	0.73 (0.391–1.87)	46.9 (30.5–71.3)
Lumbar cord	4.57 (3.0–6.98)	86.0 (53–136)	39.0 (25.2–61.5)
Brain	1.23 (1.04–1.43)	2.42 (1.03–2.41)	38.1 (29.7–54.4)
Spleen	27.0 (20.4–35.1)	0.07 (0.041–0.26)	30.6 (19.5–47.5)
Lung	18.3 (14.9–22.8)	0.92 (0.54–1.76)	23.1 (14.9–36.3)
**Linezolid**			
CSF	0.39 (0.28–0.55)	1.25 (0.86–1.87)	68.9 (47.2–94.0)
Meninges	0.30 (0.22–0.40)	0.54 (0.31–0.92)	53.2 (35.8–82.6)
Spinal cord	0.44 (0.33–0.63)	1.22 (0.89–1.63)	84.2 (62.4–98.1)
Brain	0.15 (0.10–0.20)	0.84 (0.53–1.31)	57.0 (38.2–83.2)
Spleen	1.14 (0.87–1.44)	0.99 (0.67–1.43)	59.0 (40.7–88.8)
Lung	1.06 (0.80–1.34)	0.68 (0.40–1.10)	50.6 (33.7–82.1)

[a] Values in parentheses represent the 95% confidence interval, computed using sampling importance resampling (SIR) on the final models.

[b] The estimate of the additive component of the residual unexplained variability did not differ significantly from its lower boundary of 20% of the LLOQ and was therefore fixed to this value (bedaquiline and rifampicin, 0.002 mg/L; rifabutin and linezolid, 0.001 mg/L).

REFERENCES

1. Wijk M, Wasmann RE, Jacobson KR, Svensson EM, Denti P. 2025. A Pragmatic Approach to Handling Censored Data Below the Lower Limit of Quantification in Pharmacokinetic Modeling. CPT Pharmacometrics Syst Pharmacol 14:1042–1049.
